# OCA-B promotes pathogenic maturation of stem-like CD4^+^ T cells and autoimmune demyelination

**DOI:** 10.1172/JCI187862

**Published:** 2025-04-29

**Authors:** Erik P. Hughes, Amber R. Syage, Elnaz Mirzaei Mehrabad, Thomas E. Lane, Benjamin T. Spike, Dean Tantin

**Affiliations:** 1 Department of Pathology,; 2 Huntsman Cancer Institute, and; 3 Department of Oncological Sciences, University of Utah School of Medicine, Salt Lake City, Utah, USA.; 4 School of Computing, University of Utah, Salt Lake City, Utah, USA.; 5 Department of Neurobiology and Behavior, School of Biological Sciences, University of California, Irvine, California, USA.

**Keywords:** Autoimmunity, Immunology, Multiple sclerosis, T cells

## Abstract

Stem-like T cells selectively contribute to autoimmunity, but the activities that promote their pathogenicity are incompletely understood. Here, we identify the transcription coregulator OCA-B as a driver of the pathogenic maturation of stem-like CD4^+^ T cells to promote autoimmune demyelination. Using 2 human multiple sclerosis (MS) datasets, we show that *POU2AF1*, the gene encoding OCA-B, is elevated in CD4^+^ T cells from patients with MS. We show that T cell–intrinsic OCA-B loss protects mice from experimental autoimmune encephalomyelitis (EAE) while preserving responses to viral CNS infection. In EAE models driven by antigen re-encounter, OCA-B deletion nearly eliminates CNS infiltration, proinflammatory cytokine production, and clinical disease. OCA-B–expressing CD4^+^ T cells of mice primed with autoantigen express an encephalitogenic gene program and preferentially confer disease. In a relapsing-remitting EAE model, OCA-B loss protects mice specifically at relapse. During remission, OCA-B promotes the expression of *Tcf7*, *Slamf6*, and *Sell* in proliferating CNS T cell populations. At relapse time points, OCA-B loss results in both the accumulation of an immunomodulatory CD4^+^ T cell population expressing *Ccr9* and *Bach2*, and loss of proinflammatory gene expression from Th17 cells. These results identify OCA-B as a driver of pathogenic CD4^+^ T cells.

## Abstract 2

Hughes et al. show that the transcriptional coregulator OCA-B promotes pathogenic maturation of stem-like CD4+ T cells and autoimmunity in mouse models of multiple sclerosis.

## Graphical abstract

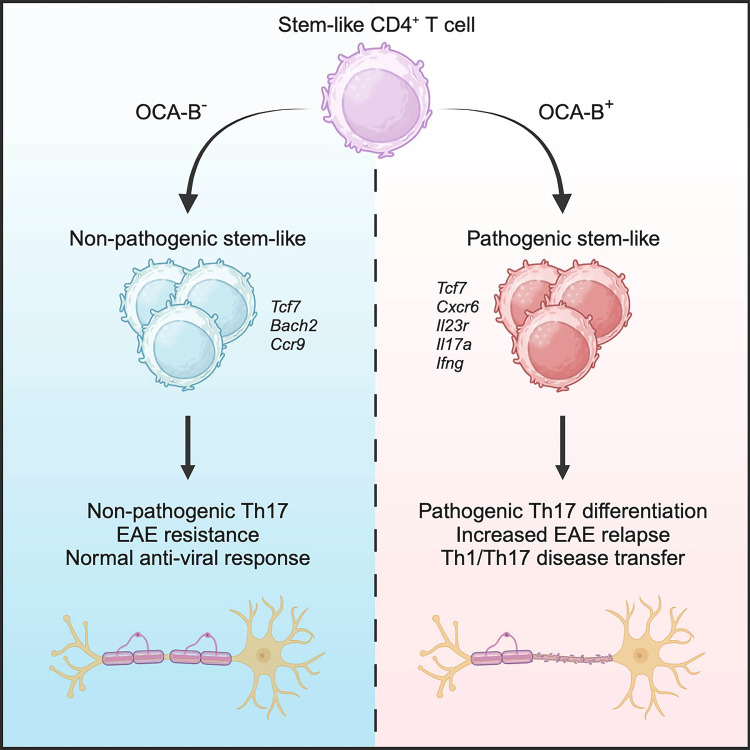

## Introduction

There are 2.8 million individuals worldwide with multiple sclerosis (MS), with almost 1 million cases occurring in the United States (1). Despite recent advances, the development of new therapies that block MS while sparing mechanisms that prevent infection and viral recrudescence remains a critical goal. GWAS have identified the *HLA* loci as the most important determinant of MS pathogenesis, emphasizing the impact of T cells (2, 3). The association with the MHC class II locus in particular indicates that myelin-specific CD4^+^ T cells are key regulators of disease.

The predominant preclinical model of MS is experimental autoimmune encephalomyelitis (EAE), in which laboratory animals are immunized with myelin proteins or peptides to activate antigen-specific CD4^+^ T cells that infiltrate the CNS and mediate disease. EAE within the C57BL/6 mouse background establishes chronic disease in which Th1 and Th17 effector CD4^+^ T cell subsets are pathogenetic mediators (4, 5). After accessing the CNS, these cells secrete IFN-γ, IL-17, GM-CSF, and other inflammatory mediators and chemoattractants γ that recruit the cell types responsible for driving demyelination (6–8).

Myelin-reactive T cells from the blood of patients with MS show stem/memory-like properties, with reduced need for costimulation (9–12). Consistent with these findings, autoreactive and bystander memory-phenotype CD4^+^ T cells preferentially transfer demyelinating disease in mouse models (13–15). Although these studies have identified vital CD4^+^ T cell populations in EAE, further research on the mechanisms sustaining these populations and driving their pathogenic activity is required to develop more precise therapies.

Oct1, a POU domain transcription factor encoded by *Pou2f1*, has been linked to autoimmune disease. GWAS studies have identified Oct1 binding site polymorphisms associated with a predisposition for autoimmune diseases, including MS (16–19). In CD4^+^ T cells, Oct1 promotes the development of both CD4^+^ memory (20) and EAE pathogenesis and demyelination (21). In contrast, Oct1 loss preserves immune responses to the neurotrophic viral infection (21). These observations highlight a promising pathway that, when targeted, may limit autoimmune CNS demyelination without the broad immune suppression elicited by many current therapeutics. However, Oct1 is widely expressed and regulates both embryonic development and somatic stem cell function (22–24), making it a poor therapeutic target.

The lymphoid-restricted transcription cofactor OCA-B regulates transcription by docking with Oct1 and, in B cells, an additional related factor, Oct2 (25). In B cells, OCA-B is essential for B cell maturation and germinal center formation (26–28). In contrast to Oct1, OCA-B whole-body knockouts are viable and fertile (28). Polymorphisms in the *OCAB* (*POU2AF1*) locus are associated with autoimmune diseases, including MS (29, 30). Although its expression is 50- to 100-fold lower in T cells compared with B cells (31), targeting either OCA-B by genetic deletion in T cells or its downstream functions with a membrane-permeable peptide mimic suppresses type-1 diabetes (32). OCA-B interacts with target genes such as *Il2*, *Ifng*, *Il21*, *Csf2* (*Gmcsf*), *Tnfrsf4* (*Ox40*), *Icos*, and *Ctla4*, but is dispensable for their expression after primary activation of T cells. Instead, OCA-B regulates these targets under a narrow range of conditions, such as after secondary stimulation in culture (20). Many of these targets are directly implicated in MS pathogenesis (6–8, 33–39). OCA-B controls expression of *Tbx21* (*Tbet*), which encodes a key regulator of Th1 effector responses but also promotes Th17 differentiation and may interact with RORγt, a master regulator of Th17 cells (20, 40, 41). In the context of MS, the only prior OCA-B work we are aware of used whole-body knockouts and showed partial protection in chronic C57BL/6 EAE models (41). Cumulatively, these findings indicate OCA-B plays poorly defined but possibly important roles in MS pathogenesis.

Here, we show that OCA-B within the T cell compartment drives EAE by promoting pathogenic maturation of stem-like CD4^+^ T cells. In C57BL/6 mice, T cell OCA-B loss substantially protects mice from chronic EAE while preserving responses to the John H. Muller strain of murine hepatitis virus (JHMV), a neurotrophic coronavirus. Using passive transfer assays in which reactive T cells are primed with antigen in donor mice, polarized in culture, and then re-encounter antigen in naive recipients, we show that OCA-B is critical for the infiltration of pathogenic cells into the CNS and the manifestation of clinical disease. Using an OCA-B reporter mouse, we find that OCA-B expressing CD4^+^ T cells taken from mice primed with myelin oligodendrocyte glycoprotein (MOG) peptide preferentially display pathogenic stem-like gene expression patterns and preferentially transfer demyelinating disease, highlighting OCA-B as both a marker and promoter of encephalitogenic autoimmune CD4^+^ T cell activity. Intriguingly, T cell conditional OCA-B knockout in EAE models on the autoimmune-prone NOD background, which develop relapsing-remitting MS (RRMS) (42), specifically protects against relapse. Single cell transcriptomic analysis of CD3ε^+^, CNS-infiltrating T cells at remission and relapse indicates that OCA-B promotes disease relapse through control of stem-like T cell populations, driving them to pathogenic Th17 differentiation. Cumulatively, these findings mark OCA-B as a critical mediator of the pathogenic maturation of autoreactive T cells and a promising potential therapeutic target.

## Results

### Elevated expression of OCAB in CD4^+^ T cells from secondary-progressive human MS lesions.

**Figure 1:**
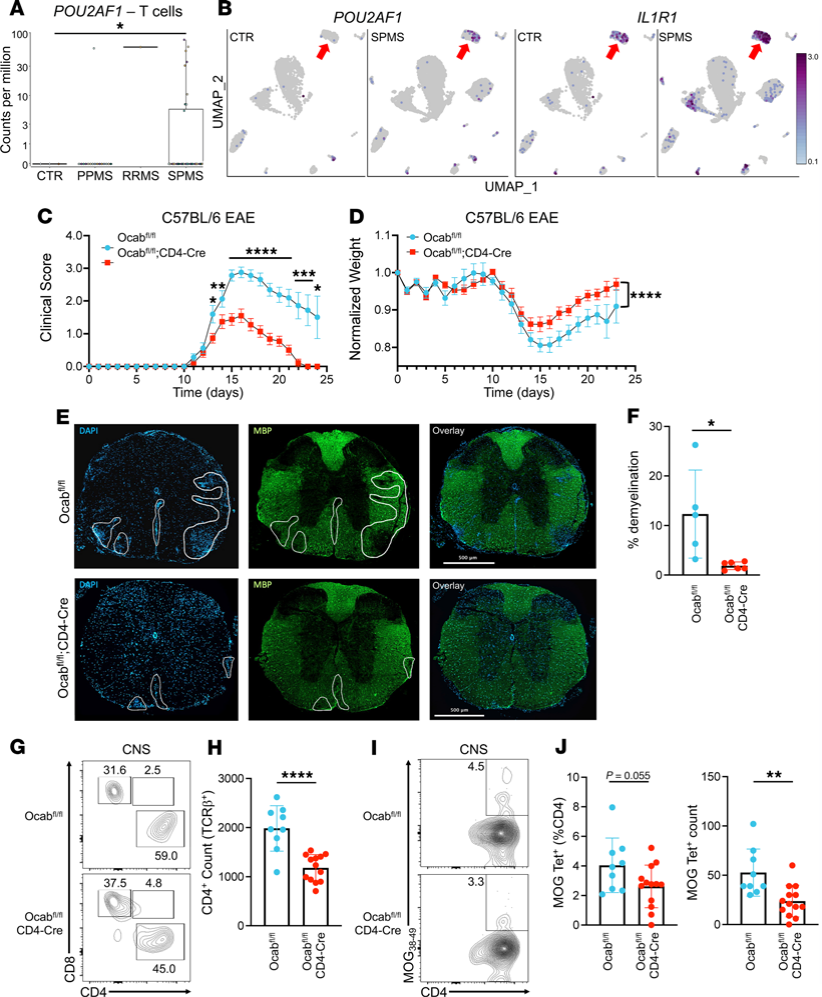
OCA-B loss protects animals from chronic EAE. (**A**) CD4^+^ T cell nuclei from single-nucleus RNA-Seq data of patients with primary-progressive (PPMS), RRMS, or SPMS lesions and from control participants (43) were identified and tested in silico for *POU2AF1* expression, which is displayed as counts per million. Significance was ascribed by Welch’s *t* test. (**B**) The CD4^+^ T cell nuclei were reclustered and displayed as UMAP feature plots showing *POU2AF1* (left) and *IL1R1* (right) expression in control (CTR) and SPMS brain tissue. Red arrow highlights a likely pathogenic CD4^+^ population expressing high levels of *IL1R1*. (**C**) *Ocab^fl/fl^* (*n* = 16) and *Ocab^fl/fl^*;CD4-Cre (*n* = 22) mice were injected with MOG_35–55_ peptide in CFA and pertussis toxin to induce EAE. Clinical scores were evaluated after EAE induction to determine disease progression. Significance was ascribed by multiple 2-tailed Student’s *t* tests. (**D**) Animal weights were recorded to evaluate weight loss as a measurement of disease progression. Significance was ascribed by 2-way ANOVA. (**E**) Representative DAPI/MBP immunostaining of thoracic spinal cord sections taken from mice 15 days after EAE induction. Areas of demyelination are marked by decreased MBP staining (green) and often coincide with increased cellular infiltration (blue). (**F**) Quantification of demyelination in *Ocab^fl/fl^* (*n* = 5) and *Ocab^fl/fl^*;CD4-Cre mice (*n* = 6). Significance was ascribed by 2-tailed Student’s *t* test. (**G**) Brain and spinal cords were isolated on day 15 of EAE and analyzed by spectral cytometry. Representative flow cytometry plots of CD45^+^TCRβ^+^ CNS infiltrating T cells showing frequency of CD4^+^ and CD8^+^ T cells. (**H**) Quantification of the number of CNS infiltrating CD4^+^ T cells. *Ocab^fl/fl^* (*n* = 9) and *Ocab^fl/fl^*;CD4-Cre (*n* = 13). Significance was ascribed by 2-tailed Student’s *t* test. (**I**) Representative plots showing frequency of MOG_38-49_ tetramer–positive CD4^+^ T cells. (**J**) Quantification of the frequency and count of MOG_38-49_ tetramer-positive CD4^+^ T cells. *Ocab^fl/fl^* (*n* = 9) and *Ocab^fl/fl^*;CD4-Cre (*n* = 13). Significance was ascribed by 2-tailed Student’s *t* test. For clinical scores and normalized weights, values represent mean ± SEM. All other values represent mean ± SD. *P ≤ 0.05, **P ≤ 0.01, ***P ≤ 0.001, and ****P ≤ 0.0001.

To determine if the expression of the gene encoding OCA-B is elevated in human MS samples, we mined high-throughput, single-nucleus RNA-Seq data of glial and immune cells from a recent study that used single-nucleus RNA-Seq to evaluate gene expression in brain tissue from 54 patients with MS and 26 control participants without MS (43). The 1 RRMS sample had far higher *POU2AF1* expression compared with the control (Figure 1A). There were more primary-progressive MS samples; interestingly, only 1 sample had detectable expression of POU2AF1, perhaps reflecting imperfections in diagnoses. Most dramatically, a subset of secondary-progressive (SPMS) samples showed significantly elevated OCA-B expression (Figure 1A). We then reclustered CD4^+^ cell nuclei from this dataset. Uniform manifold approximation and projection (UMAP) feature plots revealed that the elevated *POU2AF1* expression found in T cell nuclei of patients with SPMS was confined to 1 specific CD4^+^ cluster that also expresses the pathogenic marker *IL1R1* (Figure 1B). This cluster was also marked by the expression of the T cell activation marker *CD44* and the transcription factors *LEF1*, *ETS1*, *BCL6*, and *MAF* (Supplemental Figure 1A; supplemental material available online with this article; https://doi.org/10.1172/JCI187862DS1). To assess if elevated *POU2AF1* expression was restricted to CNS-infiltrating T cells, we analyzed a recent bulk RNA-Seq dataset (44) comparing peripheral-blood memory T effector cells (i.e., CD4^+^CD25^low/neg^CD127^hi^CD45RO^+^) from 20 patients with RRMS and 20 healthy control participants. Similar to CNS T cells, *POU2AF1* expression in peripheral CD4^+^ T cells was elevated in patients with MS compared with control participants without MS (Supplemental Figure 1B). These findings identify elevated *OCAB* mRNA expression in T cells from patients with MS across multiple datasets.

### OCA-B promotes chronic EAE in C57BL/6 mice.

To understand the impact of OCA-B in T cell mediated autoimmunity, we used an *Ocab* conditional allele crossed to the CD4-Cre driver, which efficiently deletes OCA-B in T cells (32). We induced EAE in C57BL/6 female *Ocab^fl/fl^*;CD4-Cre mice and littermate *Ocab^fl/fl^* controls through s.c. injection of MOG_35–55_ peptide in CFA followed by i.p. injections of *Bordetella pertussis* toxin. *Ocab^fl/fl^*;CD4-Cre mice had reduced chronic EAE clinical scores and weight loss compared with *Ocab^fl/fl^* (Figure 1, C and D). Spinal cords were taken at peak of disease (day 15) for immunofluorescence and histological analyses. Myelin basic protein (MBP) immunofluorescence showed a significant decrease in demyelinated white matter in spinal sections from Ocab^fl/fl^;CD4-Cre mice compared with littermate Ocab^fl/fl^ controls, and DAPI staining showed simultaneously decreased immune cell infiltration (Figure 1, E and F). Ocab^fl/fl^;CD4-Cre mice also had a significant decrease in demyelination as measured by luxol fast blue (LFB) in combination with H&E staining (Supplemental Figure 2, A and B). Flow cytometric profiling at peak disease showed decreased CD4^+^ T cell CNS infiltration in *Ocab^fl/fl^*;CD4-Cre mice; however, no differences were observed in other cell populations, anergic T cells (CD4^+^FR4^+^CD73^+^), or in the relative frequencies of the proinflammatory cytokines IFN-γ, IL-17, GM-CSF, or CD44 (Figure 1, G and H, and Supplemental Figure 2, C–H).

We studied MOG-specific CD4^+^ T cells using MHC class II (I-A^b^) MOG_38-49_ tetramers. At peak disease, *Ocab^fl/fl^*;CD4-Cre mice had a trending reduction in MOG tetramer-specific cell frequency (*P* = 0.055) and a significant reduction in numbers (*P* = 0.003) compared with controls (Figure 1, I and J). These results indicate the protection conferred by OCA-B loss in T cells is marked by modest decreases in MOG-specific CD4^+^ T cells within the CNS, resulting in fewer pathogenic T cells driving demyelination.

### OCA-B is essential for EAE mediated by adoptive transfer of Th1- and Th17-polarized T cells.

MS is driven by autoreactive CD4^+^ Th1 and Th17 cells within the CNS that recruit CD8^+^ T cells, macrophages, microglia, and neutrophils, which together mediate white-matter damage (45). To investigate the impact of OCA-B on autoimmune Th1 and Th17 populations, we used adoptive-transfer EAE models with female *Ocab*^fl/fl^;CD4-Cre and *Ocab*^fl/fl^ littermate controls. Mice were primed with MOG_35–55_ in CFA for 14 days. After MOG priming, CD4^+^ T cells were purified from the lymph nodes of primed experimental or control donors (Supplemental Figure 3A). No differences between genotypes in CD4^+^ T cell viability, count, or effector cytokine (IFN-γ and IL-17) expression were observed (Supplemental Figure 3, B–E).

**Figure 2:**
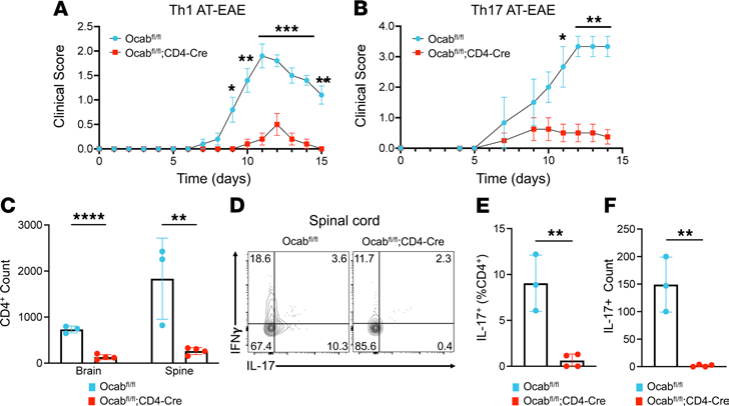
OCA-B promotes Th1 and Th17 adoptive-transfer EAE through recall response. (**A** and **B**) *Ocab^fl/fl^* and *Ocab^fl/fl^*;CD4-Cre mice were primed with MOG_35–55_ peptide in CFA for 10–14 days. Cells from the spleens and lymph nodes of primed mice were isolated and cultured for 4 days in Th1 or Th17 polarizing conditions. Th1- or Th17-polarized cells (*n* = 3.0 × 10^6^) were injected i.p. into C57BL/6 wild-type recipient mice (Th1: *n* = 5 *Ocab^fl/fl^*; *n* = 5 *Ocab^fl/fl^*;CD4-Cre) (Th17: *n* = 3 *Ocab^fl/fl^*, *n* = 4 *Ocab^fl/fl^*;CD4-Cre). Clinical scores were assessed for 14–15 days. Significance was ascribed by multiple 2-tailed Student’s *t* tests. (**C**) At Th17 adoptive-transfer EAE endpoint, brains and spinal cords were analyzed by flow cytometry. Quantification of brain- and spinal cord–infiltrating CD4^+^ T cells after Th17 adoptive transfer. Significance was ascribed by 2-tailed Student’s *t* test. (**D**) Representative flow cytometry plots showing the frequency of IFN-γ– and IL-17–expressing CD4^+^ T cells within the spines of Th17-recipient mice. (**E**) Quantification of IL-17 frequency in spinal cord–infiltrating CD4^+^ T cells. Significance was ascribed by 2-tailed Student’s *t* test. (**F**) Quantification of the total count of IL-17^+^ CD4^+^ T cells within the spinal cord of recipient mice. Significance was ascribed by 2-tailed Student’s *t* test. For clinical scores, values represent mean ± SEM. All other values represent mean ± SD. *P ≤ 0.05, **P ≤ 0.01, ***P ≤ 0.001, and ****P ≤ 0.0001.

To investigate if OCA-B expression affects Th1- or Th17-mediated adoptive transfer EAE, CD4^+^ T cells purified from donors were polarized in vitro into Th1 and Th17 cells, as previously described (46, 47). Via i.p. injection, 3 × 10^6^ Th1- or Th17-polarized CD4^+^ cells were transferred into naive, age-matched, wild-type, male C57BL/6 recipients. EAE onset in mice receiving either Th1- or Th17-polarized control cells was rapid and severe (Figure 2, A and B, and Supplemental Figure 3, F and G). In contrast to control cells, and in contrast to the mild protection observed with OCA-B deficiency in standard C57BL/6 EAE, mice adoptively transferred with T cells lacking OCA-B were almost completely protected from clinical disease regardless of their polarization state (Figure 2A and Supplemental Figure 3, F and G). Interestingly, the ability to initially polarize CD4^+^ T cells into Th1 and Th17 effectors in vitro was only minimally affected by OCA-B loss (Supplemental Figure 3, H–J), whereas cells taken from the CNS of engrafted recipient mice at day 15 not only showed decreased CNS infiltration but also reduced IL-17 expression (Figure 2, C–F). This effect appeared to be specific to IL-17, because no significant difference in IFN-γ was observed (Supplemental Figure 3K). These results indicate a central role for OCA-B in promoting robust neuroinflammatory T cell responses upon antigen re-encounter in adoptive-transfer EAE models.

### OCA-B is dispensable for neurotropic viral clearance.

**Figure 3:**
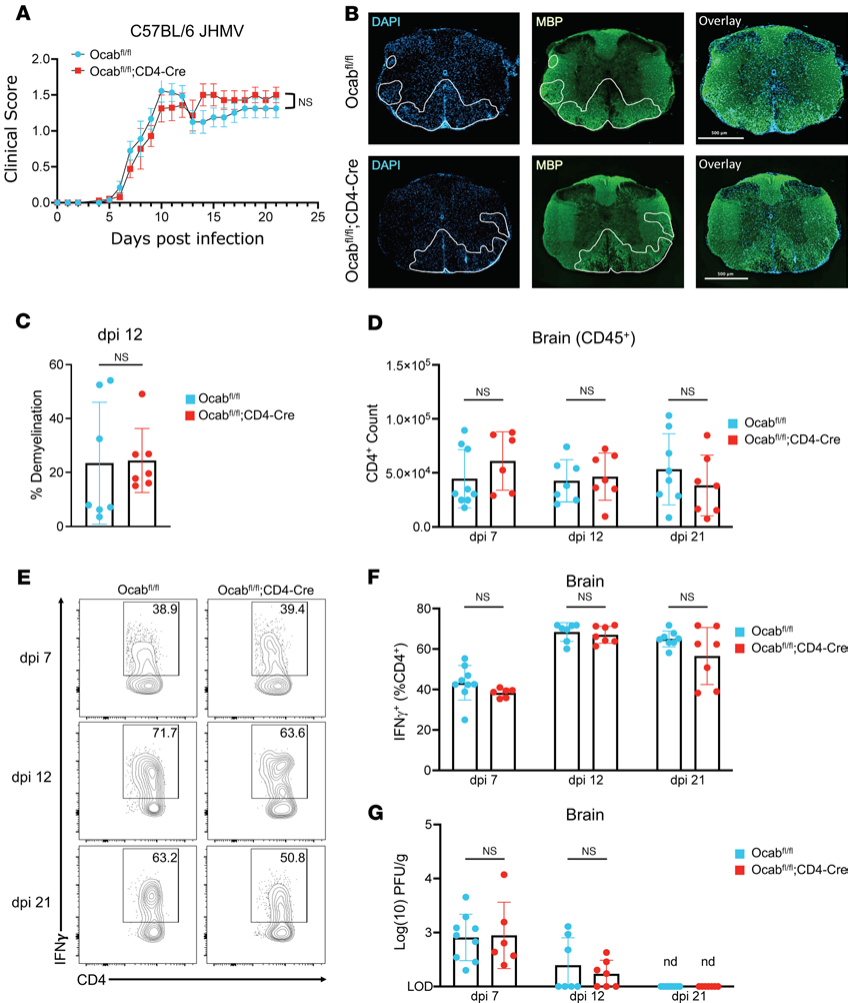
OCA-B is dispensable for T cell response to CNS infection with a neurotropic virus. (**A**) *Ocab^fl/fl^* (*n* = 19) and *Ocab^fl/fl^*;CD4-Cre (*n* = 17) mice were injected intracranially with 1500 PFU JHMV. Clinical scores were recorded for 21 dpi. Significance was ascribed by Multiple 2-tailed student’s *t* tests. (**B**) Representative DAPI/MBP immunofluorescence staining of spinal cord sections at 12 dpi. Demyelinated areas are marked by decreased MBP staining (green) and often coincide with increased cellular infiltration (blue). (**C**) Quantification of percent demyelination in spinal cord sections (*n* = 7 per group). Significance was ascribed by 2-tailed Student’s *t* test. Half-brains were taken at 7, 12, and 21 dpi for flow cytometric and viral titer analysis. (**D**) Quantification of CD4^+^ T cell counts within the half-brains at 7, 12, and 21 dpi. *Ocab^fl/fl^* (*n* = 7–9/time point) and *Ocab^fl/fl^*;CD4-Cre (*n* = 6–7/time point). Significance was ascribed by 2-tailed Student’s *t* test. (**E**) Representative plots showing the frequency of IFN-γ–expressing CD4^+^ T cells at 7, 12, and 21 dpi. (**F**) Quantification of frequency of IFN-γ–expressing CD4^+^ T cells. *Ocab^fl/fl^* (*n* = 7–9/time point) and *Ocab^fl/fl^*;CD4-Cre (*n* = 6–7/time point). Significance was ascribed by 2-tailed Student’s *t* test. (**G**) Viral titers of half-brains at dpi 7, 12, and 21 (not detected). *Ocab^fl/fl^* (*n* = 7–9/time point) and *Ocab^fl/fl^*;CD4-Cre (*n* = 6–7/time point). Significance was ascribed by 2-tailed Student’s *t* test. These data represent the combined results of 2 independent experiments. For clinical scores, values represent mean ± SEM. All other values represent mean ± SD. ns = *P* > 0.05.

To investigate if T cell OCA-B deletion affects the ability to mount immune responses and control CNS viral infection, male *Ocab*^fl/fl^;CD4-Cre and *Ocab*^fl/fl^ littermate controls were intracranially inoculated with 1500 PFU of JHMV, a β-coronavirus adapted to infect glial cells (namely, astrocytes, microglia, and oligodendrocytes) in the mouse brain and spinal cord. Inoculation with JHMV results in acute encephalomyelitis and chronic demyelination similar to autoantigen-driven EAE. Disease severity was determined by evaluating clinical scores over 21 days postinfection (dpi). *Ocab*^fl/fl^;CD4-Cre animals showed negligible differences in clinical scores compared with *Ocab*^fl/fl^ littermates (Figure 3A).

Spinal cords were taken on 12 and 21 dpi for immunofluorescence and histological analysis to investigate differences in demyelination. MBP immunofluorescence at 12 dpi showed similar demyelination and immune infiltration between Ocab^fl/fl^ and Ocab^fl/fl^;CD4-Cre spinal sections (Figure 3, B and C). Similarly, histology of Luxol fast blue–stained brain tissue at 21 dpi showed no significant differences in demyelination between OCA-B–deficient and control infected animals (Supplemental Figure 4, A and B). Flow cytometric analysis of brain tissue showed no significant differences in infiltrating CD4^+^ T cell numbers or IFN-γ expression at 7, 12, or 21 dpi (Figure 3, D–F, and Supplemental Figure 4C). Similarly, no differences in either total CD8^+^ T cell numbers or the percentage or numbers of IFN-γ–expressing CD8^+^ T cells were observed (Supplemental Figure 4, D–F). Brain hemisphere homogenates were collected on 7, 12, and 21 dpi to measure viral titers and clearance. *Ocab*^fl/fl^;CD4-Cre and *Ocab*^fl/fl^ littermate control animals had similar titers at dpi 7 and 12, and virus was undetectable at day 21 regardless of genotype (Figure 3G). This result suggests OCA-B is dispensable for baseline immune responses to and clearance of neurotropic viruses.

### OCA-B expressing CD4^+^ T cells display pathogenic stem-like properties and preferentially transfer demyelinating disease.

**Figure 4:**
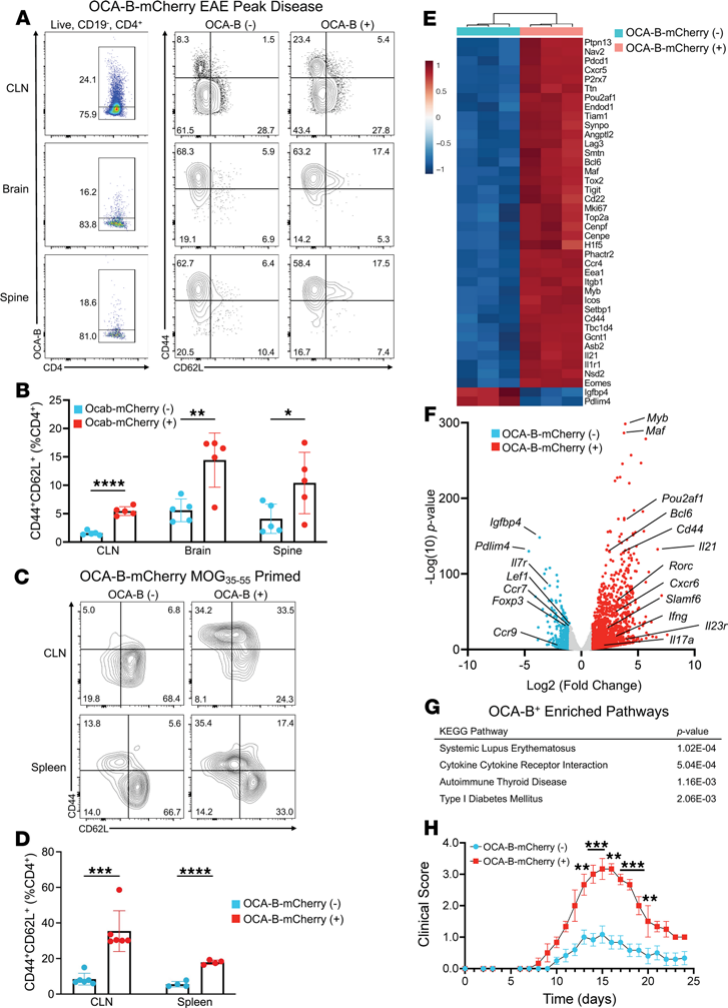
OCA-B expression marks encephalitogenic stem-like CD4^+^ T cells. (**A**) OCA-B–mCherry mice were injected with MOG_35–55_ peptide in CFA and pertussis toxin to induce EAE. Cervical lymph nodes (CLNs), brains, and spines were isolated at peak disease (day 15) and analyzed by flow cytometry for mCherry, CD44, and CD62L expression. (**B**) CD44^+^- and CD62L^+^-expressing CD4^+^ T cells were quantified based on mCherry expression. Mean values are shown from 5 biological replicates. Significance was ascribed by 2-tailed Student’s *t* test. (**C**) OCA-B–mCherry mice were injected with MOG_35–55_ peptide in CFA, and after 14 days, CD4^+^ T cells within CLNs and spleens were profiled by flow cytometry for mCherry, CD44, and CD62L expression. (**D**) Quantification of CD44 and CD62L expression of OCA-B– negative and –positive CD4^+^ T cells from MOG-primed OCA-B–mCherry mice. *n* = 4–6 biological replicates from 2 combined replicate experiments are shown. Significance was ascribed by 2-tailed Student’s *t* test. (**E**) Heatmap showing relative expression of the top 40 differentially expressed genes from OCA-B–positive and –negative CD4^+^ T cells from bulk RNA-Seq of OCA-B–positive and –negative CD4^+^ T cells from MOG_35–55_ primed OCA-B-mCherry reporter mice. (**F**) Volcano plot of differentially expressed genes between OCA-B–positive and –negative T cells (*P* < 0.05 and log2-fold change >1 data points are colored). (**G**) Gene Ontology terms associated with OCA-B–positive and –negative groups. (**H**) EAE clinical scores of C57BL/6 wild-type mice after passive transfer of OCA-B–positive or –negative Th1 polarized CD4^+^ T cells. Significance was ascribed by multiple 2-tailed Student’s *t* tests. Clinical score values represent mean ± SEM. All other values represent mean ± SD. ns = *P* > 0.05, *P ≤ 0.05, **P ≤ 0.01, ***P ≤ 0.001, and ****P ≤ 0.0001. KEGG, Kyoto Encyclopedia of Genes and Genomes.

We used a recently described OCA-B–mCherry reporter mouse (31) to evaluate OCA-B expression within CNS infiltrating T cells. EAE was induced in female C57BL/6 homozygous OCA-B-mCherry reporter mice with MOG_35–55_ and CNS-infiltrating CD4^+^ T cells assessed by flow cytometry at peak clinical disease. mCherry-positive T cells showed increased CD44 and CD62L co-expression, indicative of increased memory-like cells in the OCA-B–expressing population (Figure 4A). The association of OCA-B expression with a memory-like profile was observed in the brain, spine, and draining cervical lymph nodes (Figure 4B). Interestingly, the frequency of CD44^+^CD62L^–^CD4^+^ T cells was elevated within the OCA-B–expressing population specifically within the cervical lymph nodes (Supplemental Figure 5A).

Memory-like CD4^+^ T cells have been implicated as major orchestrators of autoimmune demyelination in mice and humans (9–12, 14, 15, 48). To see if this memory-like profile of OCA-B–expressing CD4^+^ T cells is specific to CNS infiltrating cells, we performed flow cytometry using draining lymph node and splenic T cells from MOG-primed OCA-B-mCherry reporter mice. Similar to OCA-B^+^ CNS-infiltrating cells, OCA-B–expressing CD4^+^ T cells taken from the cervical lymph node and spleen had increased CD44 and CD62L co-expression (Figure 4, C and D).

We next evaluated OCA-B–mCherry positive and negative populations by RNA-Seq of CD4^+^ T cells isolated from the lymph nodes of 14-day MOG-primed mice. CD4^+^ T cells were sorted based on mCherry expression, as well as lack of CD8, lack of CD19, expression of CD4, and viability (Supplemental Figure 5B). Approximately 1500 genes were upregulated and approximately 350 were downregulated in OCA-B–expressing cells compared with OCA-B–negative cells (Supplemental Table 1). Upregulated genes in OCA-B–expressing cells included *Cxcr6*, *Slamf6*, *Il23r*, *Rorc*, *Ifng*, *Bcl6*, and *Il17a*, and downregulated genes included *Foxp3*, *Il7r*, and *Ccr9* (Figure 4, E and F). Expression of *Tcf7* (which encodes TCF1) was high within both OCA-B–positive and –negative populations (Supplemental Table 1). Gene ontology and pathway analysis of upregulated genes show enrichment for terms associated with autoimmune diseases, including lupus, autoimmune thyroid disease, and type 1 diabetes (Figure 4G and Supplemental Table 2).

To further assess how OCA-B may transcriptionally regulate these differentially regulated genes, we analyzed our previously published Oct1/OCA-B ChIP-Seq dataset (20). After realignment to the *mm39* reference genome, Oct1 and OCA-B co-localized peaks were observed both near the transcription start site and within gene body of *Tcf7*, *Bcl6*, and *Bach2* (Supplemental Figure 5C). These findings indicate OCA-B likely directly regulates at least a subset of the differentially expressed genes identified in gene knockout experiments.

To determine if OCA-B expression can be used to prospectively identify viable pathogenic CD4^+^ T cell populations, we used OCA-B–mCherry reporter mice as donors in passive-transfer EAE. Male and female littermates (11 weeks old), homozygous for the OCA-B-mCherry reporter, were primed for 14 days with MOG_35–55_ in CFA, as previously described (47). We cultured 9 × 10^5^ mCherry-negative and -positive cells in Th1 polarizing conditions and subsequently transferred them into age-matched male C57BL/6 recipients, as previously described (14). Disease progression was measured by clinical score and normalized weight. Recipients of OCA-B–expressing mCherry-positive cells had increased clinical disease severity and weight loss compared with mCherry-negative control recipients (Figure 4H and Supplemental Figure 5D). These findings indicate OCA-B marks CD4^+^ T cells with pathogenic stem-like gene expression and can be used to prospectively identify peripheral CD4^+^ T cells with enhanced encephalitogenic properties.

### OCA-B promotes neuroinflammation in relapsing-remitting EAE models via control of stem/memory-like T cell populations.

**Figure 5:**
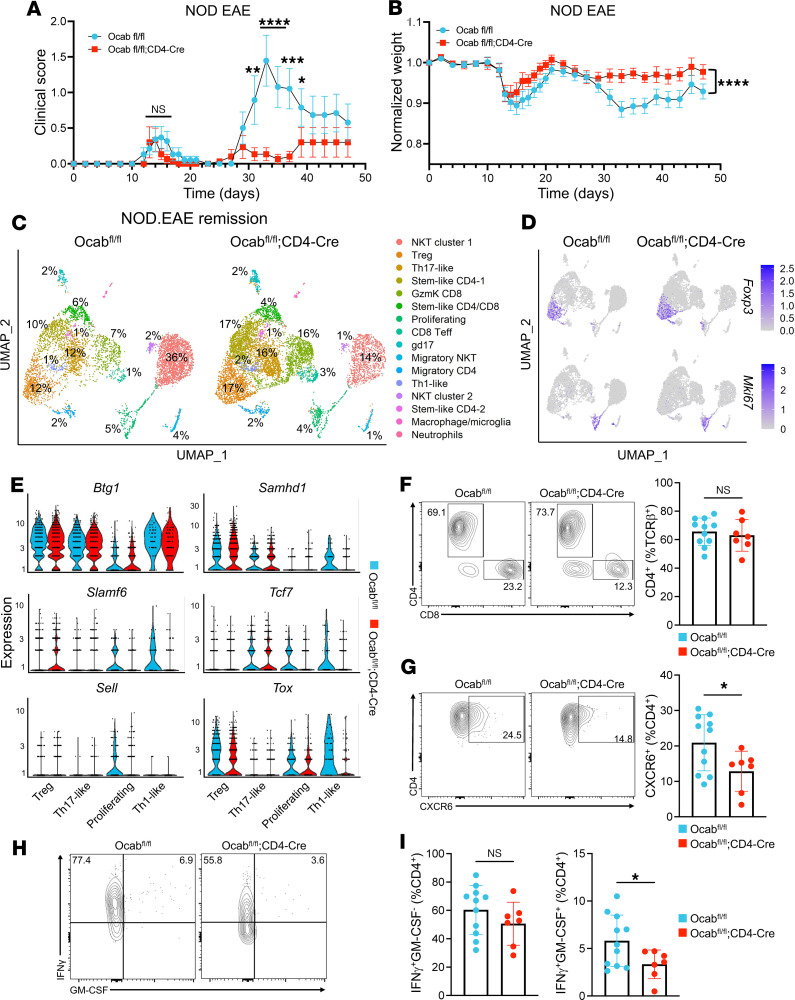
OCA-B promotes relapsing-remitting EAE through stem-like CD4^+^ T cells. (**A**) NOD.*Ocab^fl/fl^* (*n* = 19) and NOD.*Ocab^fl/fl^*;CD4-Cre (*n* = 15) mice were injected with MOG_35–55_ peptide in CFA and pertussis toxin to induce EAE. Clinical scores of animals representing relapsing-remitting disease progression are shown as a function of time. Significance was ascribed by multiple 2-tailed Student’s *t* tests. (**B**) Animal weights were recorded to determine weight loss throughout initial disease and relapse. Significance was ascribed by 2-way ANOVA. (**C**) scRNA-Seq was performed on pooled CD3ε^+^ cells isolated from the brain and spine of NOD.*Ocab^fl/fl^* (*n* = 3) and NOD.*Ocab^fl/fl^*;CD4-Cre (*n* = 4) 24 days after EAE induction (remission time point). Cell populations were plotted in a UMAP using the *Seurat* R package, and percentages are shown for each cluster. Clusters were identified through differential gene expression analysis. (**D**) Feature plots comparing the expression of *Foxp3* and *Mki67* among clusters. (**E**) Violin plots showing the expression of *Btg1*, *Samhd1*, *Slamf6*, *Tcf7*, *Sell*, and *Tox* within the Treg, Th17-like, proliferating, and Th1-like clusters. (**F**) CNS cells isolated at remission time point (day 24) were analyzed by spectral cytometry. Representative flow cytometry plots and quantification showing the frequency of CD4^+^ T cells between control and experimental groups. Significance was ascribed by 2-tailed Student’s *t* test. (**G**) Representative flow cytometry plots and quantification showing the frequency of CXCR6 expressing CD4^+^ T cells. Significance was ascribed by 2-tailed Student’s *t* test. (**H**) Representative flow cytometry plots showing the frequency of CD4^+^ T cells expressing IFN-γ and GM-CSF. (**I**) Quantification of CD4^+^ T cells expressing IFN-γ and GM-CSF. Significance was ascribed by 2-tailed Student’s *t* test. For clinical scores, data represent mean ± SEM. All other data represent mean ± SD. ns = *P* > 0.05, *P ≤ 0.05.

In contrast to the chronic form of EAE that develops in C57BL/6 mouse models, EAE on the NOD background exhibits a relapsing-remitting pattern that more closely mimics human MS (42). We induced EAE in female NOD.*Ocab*^fl/fl^;CD4-Cre and littermate control NOD.*Ocab*^fl/fl^ mice (15–18 weeks old) (32) with MOG_35–55_. Clinical scores and weights were recorded for 47 days to observe initial disease onset, relapse, and relapse recovery. No significant differences in clinical scores were observed between groups at disease onset (days 10–19). However, after the primary remission, NOD.*Ocab*^fl/fl^;CD4-Cre animals were almost completely protected against disease relapse (Figure 5A). Individual NOD mouse clinical scores are shown in Supplemental Figure 6. Normalized weights of animals also reflected the protective effect of OCA-B loss in T cells at relapse (Figure 5B).

To understand differences in CNS-infiltrating T cells underlying relapse protection at population and transcriptional levels, we performed single-cell RNA-Seq (scRNA-Seq) of CD3ε^+^ T cell isolates from CNS (brain and spinal cord) at remission (day 24) and peak relapse (day 33). Cells from 3–6 NOD.*Ocab*^fl/fl^;CD4-Cre and NOD.*Ocab*^fl/fl^ littermate controls were collected and combined for analysis. A total of 7799 (remission) and 9224 (relapse) *Ocab* knockout and 6009 (remission) and 7525 (relapse) control cells passed filtering and were used for analysis, with an average per cell read depth ranging from 90,000 to 190,000. For remission, UMAP clustering using both genotypes revealed 16 clusters corresponding to a broad T cell repertoire consisting of NKT cells (the largest cluster), γδ T cells with a Th17-like gene signature (gd17), and different CD4^+^ and CD8^+^ subsets (Figure 5C and Supplemental Figure 7A). Noteworthy clusters based on mRNA expression included proliferating (*Ki67*, *Pcna*, *Mcm2*), Th1-like (*Tbx21*, *Ifng*, *Tnf*, *Il12rb2*), and Treg (*Foxp3*, *Ctla4*) (Supplemental Figure 7, A and B, and Supplemental Table 3). Feature plots for *Foxp3* and *Mki67* are shown in Figure 5D. Additional feature plots for *Cxcr6*, *Il23r*, and *Bach2* are shown in Supplemental Figure 7C. *Bach2* was notably poorly expressed in all clusters. Notably, there was a large decrease in NKT cell numbers in knockout mice (36% control vs. 14% experimental). Within this cluster, changes in gene expression between control and conditional knockout groups were minimal. Most NKT cells from both groups had a limited inflammatory cytokine response, consistent with the lack of clinical disease at remission. The decrease in the large NKT population in the experimental group proportionally increases the percentage contribution of the other clusters; therefore, similar or decreased percentages of these cells identify reduced populations, which correlate with protection from relapse (e.g., proliferating T cells). Other clusters, such as Tregs, increased disproportionately. Differential gene expression analysis of key clusters (Treg, Th17-like, proliferating, and Th1-like) revealed increased expression of *Slamf6* in Tregs lacking OCA-B, whereas OCA-B–deficient proliferating and Th1-like cells had decreased expression of genes associated with quiescence (*Btg1* and *Samhd1*), memory/stemness (*Slamf6*, *Tcf7*, and *Sell*), and pathogenicity (*Tcf7* and *Tox* co-expression) (Figure 5E). Spectral cytometry showed no significant difference in the frequency of CNS-infiltrating CD4^+^ T cells or their expression of PD-1 between groups at remission (Figure 5F and Supplemental Figure 8, A and B). However, OCA-B–deficient CD4^+^ T cells did show a significant reduction in CXCR6 and IFN-γ/GM-CSF co-expression, indicative of reduced Th17 pathogenicity (Figure 5, G–I). These results indicate loss of OCA-B in T cells selectively reduces proliferation and differentiation of pathogenic stem/memory-like CD4^+^ T cells during disease remission.

**Figure 6:**
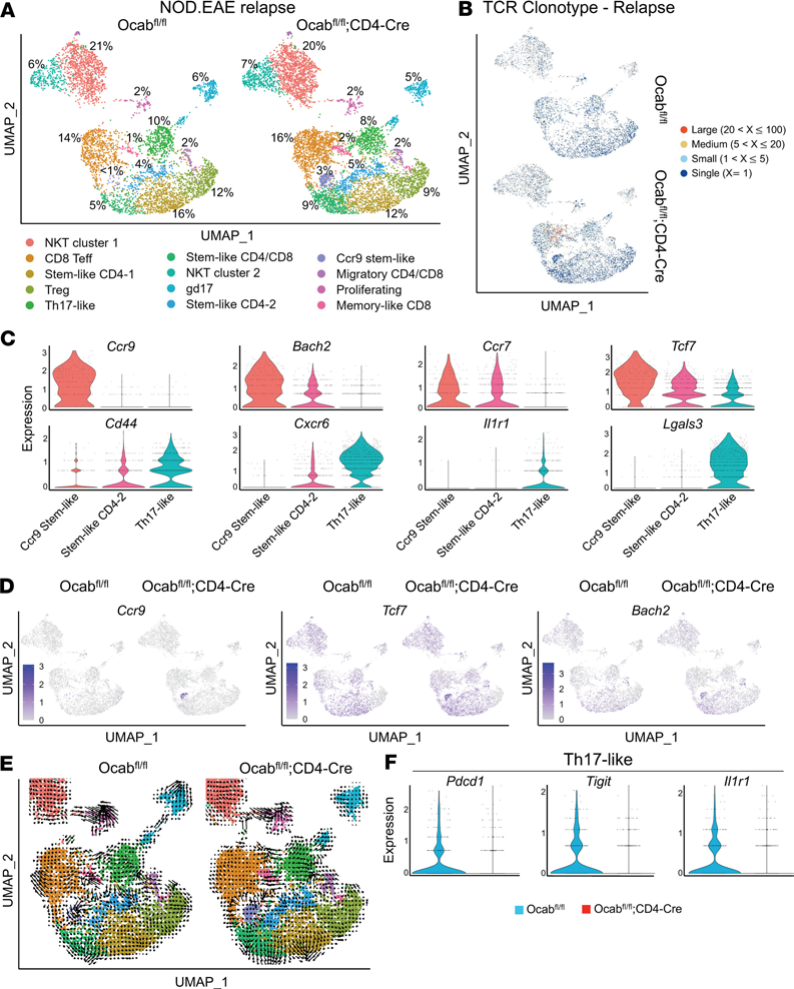
OCA-B promotes disease relapse through control of pathogenic stem-like Th17 differentiation. (**A**) scRNA and TCR sequencing were performed on pooled CD3ε^+^ cells isolated from the brain and spine of NOD.*Ocab^fl/fl^* (*n* = 5) and NOD.*Ocab^fl/fl^*;CD4-Cre (*n* = 6) mice 33 days after EAE induction (relapse time point). Cell populations were plotted in a UMAP using the *Seurat* R package and percentages are shown for each cluster. Clusters were identified through differential gene expression analysis. (**B**) UMAP TCR clonotype expansion between *Ocab^fl/fl^* and *Ocab^fl/fl^*;CD4-Cre among clusters. (**C**) Violin plots showing expression of *Ccr9*, *Bach2*, *Ccr7*, *Tcf7*, *Cd44*, *Cxcr6*, *Il1r1*, and *Lgals3* in the Ccr9 stem-like CD4, stem-like CD4-2, and Th17-like clusters. (**D**) Feature plots comparing the expression of *Ccr9*, *Tcf7*, and *Bach2* among clusters**.** (**E**) UMAPs showing RNA velocity of spliced and unspliced transcripts by experimental condition. (**F**) Violin plots showing expression of *Pdcd1*, *Tigit*, and *Il1r1* in the Th17-like cluster between control and experimental groups.

At relapse, multiple CNS-infiltrating stem-like and effector CD4^+^ and CD8^+^ control T cell populations were present (Figure 6A, Supplemental Figure 9, A and B, and Supplemental Table 4). T cell receptor (TCR) sequencing revealed an expansion of a subset of TCR clones in the OCA-B–deficient CD8 clusters, whereas most other clusters were highly polyclonal (Figure 6B). Comparing the 10 most expanded TCR clonotypes between knockouts and controls, several clonotypes were shared but differentially expanded (Supplemental Figure 9C and Supplemental Table 5). A Ccr9 stem-like cluster expressing the gut-homing chemokine receptor *Ccr9*, as well as *Ccr7*, *Bach2*, and *Tcf7*, notably appeared at relapse (Figure 6C and Supplemental Table 4). TCR clonotype analysis indicated that this population was highly polyclonal (Figure 6B). Additionally, a stem-like CD4-2 cluster expressing *Tcf7*, Ccr7, *Bach2*, *Cd44*, and low-level *Cxcr6*, and a prominent Th17-like cluster expressing *Tcf7*, *Cd44*, *Il1r1*, *Lgals3*, and higher levels of *Cxcr6* were identified (Figure 6C). The latter cluster likely contains pathogenic effector cells.

In the absence of OCA-B, NKT cell populations were normalized compared with the remission time point, and several clusters showed major changes in the percentage of cells, including most prominently within the Ccr9 stem-like cluster, which was much more prevalent (Figure 6D). Feature plots comparing OCA-B T cell–deficient mice and littermate controls for *Ccr9* and the transcription factors *Tcf7* and *Bach2* are shown in Figure 6D. The cells were also marked by the expression of *Cd4*, *Tox*, *Ccr7*, and *Ccr4*, and limited expression of *Sell* (Supplemental Figure 10A).

RNA velocity analysis of spliced and unspliced transcripts (49) identified clusters of T cells, most of which had static gene expression profiles consistent with terminal differentiation but some with differentiating or transitional gene expression (Figure 6E). Notably, OCA-B knockout cells had increased transcriptional stasis within the Ccr9 stem-like and proliferating clusters, and a decreased transcriptional directionality of Th17 cells toward gd17 cells, the latter of which express high levels of pathogenic markers, including *Lgals3*, *Cxcr6*, and *Il1r1* (Supplemental Figure 9, A and B). Pseudotime analysis identified a transcriptional capacity in the control *Ccr9*-expressing population to differentiate into pathogenic Th17-like cells, whereas cells lacking OCA-B were limited in this capacity, possibly resulting in the accumulation of these cells (Supplemental Figure 10B). These changes culminated in the Th17-like effector knockout cells expressing reduced pathogenicity-associated genes such as *Pdcd1*, *Tigit*, and *Il1r1* (Figure 6F). Together, these results suggest OCA-B mediates the transition of nonpathogenic stem-like cells to pathogenic Th17 cells, which ultimately drive disease relapse.

## Discussion

The development of therapies that inhibit autoimmunity while minimizing impacts on normal immune function remains a critical unmet goal in the field. In this study, we show that expression of mRNA encoding the transcriptional coregulator OCA-B is elevated in CD4^+^ T cells from SPMS brain tissue and from effector memory-like, peripheral-blood CD4^+^ T cells from patients with RRMS. We show that OCA-B promotes the pathogenic maturation of T cells and establishment of CNS autoimmunity, particularly episodes of relapse. Loss of OCA-B attenuates these populations in chronic EAE, in adoptive-transfer EAE, and in NOD.EAE during remission. During relapse, OCA-B T cell knockout mice are almost completely protected and accumulate a population of CD4^+^ T cells expressing *Ccr9*, *Bach2*, and *Tcf7*. OCA-B expression also marks viable pathogenic stem/memory-like CD4^+^ T cells that preferentially transfer EAE. Furthermore, the effects of modulating OCA-B are selective, because responses to infection with a neurotropic coronavirus that generates similar clinical symptoms but lacks an autoantigen were largely unaffected.

OCA-B is a member of a small family of transcription coregulators that also includes OCA-T1, OCA-T2, and IκBζ (50, 51). It becomes induced upon CD4^+^ T cell activation and docks with Oct1 to directly regulate immunomodulatory genes such as *Il2*, *Ifng*, *Il17a*, *Tbx21* (*T-bet*), and *Csf2* (*Gmcsf*). Rather than functioning as a primary activator of these genes, OCA-B is required for their robust expression upon antigen re-encounter (20, 32). Notably, unlike transcription factors such as NF-AT, AP-1 and NF-κB, Oct1 and OCA-B are dispensable for normal primary T cell responses (20). These findings predict important roles for OCA-B in CD4^+^ T cells, specifically in situations of antigen re-encounter, which is a necessary feature of autoimmunity. Conditional T cell knockout of Oct1 reduces EAE severity, with reduced proinflammatory cytokine expression and increased anergy (21). Deletion of Oct1 in T cells was dispensable for neurotropic viral clearance (21); however, the ubiquitous expression of Oct1 in adult tissue limits its translational potential. In contrast, OCA-B expression is largely confined to B and T cells, and whole-body knockout mice are viable and fertile (52). OCA-B is highly expressed in B cells and required for germinal center formation (52, 53). OCA-B is expressed in CD4^+^ T cells at levels 50- to 100-fold less than in B cells (32, 54, 55), which suggests that with proper therapeutic dosing, OCA-B could be inhibited selectively in T cells with minimal impact to the B cell compartment. Short-term administration of an OCA-B peptide mimic that inhibits OCA-B’s downstream effector functions reverses spontaneous type-1 diabetes in NOD mice (32). However, toxicity would limit the peptide mimic’s efficacy, including in EAE models. Further research is required for the development of safe and effective OCA-B inhibitors.

OCA-B whole-body knockout animals are partially protected from EAE (41); however, the known activity of OCA-B in B cells made it unclear if the protection is T cell derived. We show that OCA-B promotes EAE pathogenesis through T cells. Opportunistic infections and reactivation of latent CNS-tropic viruses are noteworthy issues for MS therapeutics that focus on global immune suppression as a therapeutic mechanism. When OCA-B T cell conditional knockout mice are challenged with the neurotropic coronavirus JHMV, there is little difference in their ability to mount immune responses and control virus replication. These data are consistent with previous findings that OCA-B loss in T cells preserves lymphocytic choriomeningitis virus acute viral infection response, instead selectively affecting memory recall responses (20, 31). This finding provides a potential therapeutic window in which targeting the low levels of OCA-B in T cells could be used to blunt autoimmunity while sparing baseline immune responses.

Unlike the significant but modest protection observed in chronic C57BL/6 EAE, OCA-B loss in adoptive transfer EAE almost completely protects recipient animals from disease. This may indicate that adoptive-transfer EAE models involve more autoantigen re-exposures, because transferred T cells must re-encounter antigen in a naive host before promoting disease. Protection was observed regardless of whether donor cells were Th1 or Th17 polarized, indicating that OCA-B functions through a mechanism that parallels rather than enforces the Th1/Th17 paradigm. Therefore, we sought other mechanisms that might explain why OCA-B T cell knockouts are so strongly protected.

Autoimmune pathogenesis and maintenance have been increasingly associated with stem- or progenitor-like T cell subpopulations (56, 57). Recent findings indicate an emerging role for stem/memory-like T cells in promoting autoimmunity (48, 58–60). For example, myelin-specific CD4^+^ T cells in patients with MS show signs of a previously activated, memory-like phenotype compared with healthy controls (9, 10, 12). Furthermore, memory-like Th1 and Th17 cells are increased in the peripheral blood of patients with MS and display increased pathogenicity compared with conventional Th17 cells (61). Similarly, in EAE models, peripheral central memory-like CD4^+^CD44^hi^CD62L^hi^ T cells confer increased disease severity upon transfer (14). Both Oct1 and OCA-B promote the functionality of long-lived CD4^+^ memory T cells following antigenic rechallenge (31, 62). An OCA-B–mCherry reporter mouse allele, which effectively labels memory progenitor CD4^+^ T cells during acute infection (31), also labels CD4^+^ T cells that preferentially confer EAE. OCA-B^hi^ cells taken from the CNS at peak EAE selectively express stem/memory markers. Further profiling OCA-B^hi^ CD4^+^ T cells suggests that OCA-B promotes the transition of nonpathogenic stem-like cells to a pathogenic state associated with markers such as *Cxcr6*, *Slamf6*, *Ifng*, and *Il17a*. Elevated *Pou2af1* (*Ocab*) transcripts are also found in pathogenic Th17 cell populations (63). The connection between OCA-B expression and pathogenic memory/stem-like T cells likely has not been appreciated before because OCA-B is expressed at levels too low to be reproducibly captured with many current scRNA-Seq technologies.

Relapsing-remitting EAE models have been developed using the Swiss Jim Lambert and autoimmune-prone NOD backgrounds (42, 64). In NOD mice, MOG_35–55_ EAE has been described as relapsing-remitting with minimal initial disease that increases in severity upon relapse (65, 66). Using this model with the conditional *Pou2af1* allele backcrossed to the NOD background (32), we found that OCA-B T cell loss minimally affects initial clinical presentation; instead it protects animals specifically at relapse. The finding that OCA-B loss in conventional C57BL/6 EAE results in partial protection intermediate between the initial and relapse phases of NOD.EAE suggests that chronic C57BL/6 EAE may not model either initial presentation or relapse accurately but instead represents a superposition of the 2. Nevertheless, there are caveats associated with the NOD.EAE model, including poor characterization relative to the C57BL/6 and Swiss Jim Lambert models, relatively weak clinical scores, lack of temporal synchrony between replicate animals after the first relapse, and lack of underlying progression (42, 67). Although we found that *POU2AF1* transcripts are elevated in T cells from lesions of patients with SPMS, more work is necessary to confirm selectivity for OCA-B in promoting patient relapse and underlying progression.

Single-cell RNA expression profiling at remission identifies a substantial loss of *Slamf6*, *Tcf7* (encoding TCF1), and *Sell* from proliferative stem-like CD4^+^ T cells in the OCA-B–deficient condition. Although most research on TCF1-expressing stem-like T cells focuses on CD8^+^ populations with the capacity for self-renewal and differentiation into effector cells, analogous descriptions have been made for CD4^+^ populations (48, 68, 69). Indeed, autoimmune CD4^+^ T cells expressing these memory/stem-like markers have been reported as an encephalitogenic reservoir that plays a key role in EAE pathogenicity (48). In the same study, bulk RNA-Seq associated OCA-B expression within this stem-like reservoir (48).

scRNA-Seq at relapse identified multiple emergent populations of effector T cells compared with at remission. Notably, there was also an accumulation of a unique population of CD4^+^ cells in OCA-B T cell–deficient mice. These cells are conspicuously marked by expression not only of *Ccr4* but also of *Ccr9*, which encodes a gut-homing chemokine receptor. CCR9^+^ memory-like CD4^+^ T cells have been linked to MS and EAE protection (70). The cells also express *Bach2*, a transcription regulator that recently was associated with non-pathogenic, immunomodulatory Th17-polarized cells in the context of C57BL/6 EAE (71). *Bach2* expression was largely concentrated in the CCR9 stem-like cluster, which is more numerous in the knockout condition. Consistently, in bulk RNA-Seq, *Bach2* mRNA was upregulated in OCA-B^–^ cells (*P* < 10^–13^), although with a small (+1.74) fold change (Supplemental Table 1). Similarly, recent work has associated *Pou2af1* (*Ocab*) with pathogenic Th17/Th1 cells compared with the nonpathogenic *Bach2*-expressing cells (71). In B cells, OCA-B regulates *Bach2* (72); however, the interplay between OCA-B and Bach2 and how it influences the pathogenic maturation of Th17 cells merit additional research. Velocity and pseudotime analysis indicates that these cells are normally transcriptionally inclined to progress toward an highly pathogenic Th17 population but that progression stalls in mice whose T cells lack OCA-B. Consistently, the Th17 effector population in OCA-B T cell knockout mice lacks expression of key genes such as *Il1r1* and *Pdcd1*, both of which are associated with pathogenicity in CNS-infiltrating Th17 cells (48, 73). The cumulative alterations in these populations in OCA-B knockouts likely explains why these mice are strongly protected against disease relapse. In conclusion, this study indicates a prominent role for OCA-B in driving stem-like CD4^+^ T cell populations toward a pathogenic state while playing a minimal role in acute antiviral responses to a CNS infection.

## Methods

### Sex as a biological variable.

The objective of this study was to determine the role of the transcriptional regulator OCA-B in demyelinating disease. OCA-B T cell conditional knockout mice and OCA-B reporter mice were used in multiple mouse-demyelinating-disease models to evaluate the impact of T cell OCA-B expression on clinical disease manifestation and the phenotypes of neuroinflammatory cells, particularly T cells. Male and female animals were used for experiments except in active EAE experiments, for which the better model of spinal cord demyelination and inflammation is female mice.

### Analysis of a publicly available human control and MS single-nuclei RNA-Seq dataset.

Initial evaluation of *POU2AF1* expression in T cells was conducted in the interactive web browser created by the authors of the dataset available at https://malhotralab.shinyapps.io/MS_broad/ (43). Briefly, *POU2AF1* differential gene expression was filtered to show T cell gene expression by diagnosis with a minimal threshold cutoff of 10 cells/sample. Cleaned, aligned matrix files were then downloaded from https://zenodo.org/records/8338963 and subsequently processed by using the 10X Genomics CellRanger pipeline and further analyzed using the 10X Genomics Loupe Browser 7 software. A total of 19,906 CD4^+^ cells were then subclustered and evaluated for *POU2AF1* and *IL1R1* gene expression by diagnosis.

### Chronic C57BL/6 EAE.

Female C57BL/6 littermate animals (11–12 weeks old) were used for all experiments. For adoptive-transfer experiments using the OCA-B–mCherry reporter allele (31), 11-week-old female mice were used for chronic EAE and 9-week-old male mice were used as MOG_35–55_-primed adoptive-transfer donors. EAE was induced on day 0 by two 100 μL s.c. injections into both hind flanks of 0.5 μmol/mL MOG_35–55_ peptide (University of Utah Peptide Core) emulsified in CFA, which was composed of incomplete Freund’s adjuvant (77145, Thermo Fisher) together with 4 mg/mL heat-killed *Mycobacterium* *tuberculosis* (DF3114338, Fisher Scientific). Animals were given two 400-ng i.p. injections of *B*. *pertussis* toxin (181, List Biological Laboratories) on days 0 and 2.

### Clinical scoring.

EAE clinical scores were determined using a 0–5 scale with 0.5 scoring intervals: 0.5, tip of tail is limp or tail muscle strain observed; 1, completely limp tail; 1.5, completely limp tail and at least 1 hind limb consistently falls through wire rack when animal is dropped; 2, completely limp tail and 1 hind leg drags without movement beyond hip; 2.5, completely limp tail and both hind limbs drag without movement beyond hip; 3, complete hind limb paralysis; 3.5, complete hind limb paralysis with flat-laying hind quarters and hump in upper torso; 4, complete hind limb and partial forelimb paralysis, minimal movement around cage; 4.5, complete hind limb and partial forelimb paralysis, no movement around cage; 5, seizure or death. Animals scoring a 4 or higher for longer than 48 hours were euthanized and scored 5 for the remainder of the experiment. JHMV clinical disease was evaluated using a previously described 0–4 point scale (74).

### Immunofluorescence and histology.

Spinal columns were isolated from C57BL/6 *Ocab^fl/fl^* and *Ocab^fl/fl^*;CD4-Cre animals after EAE or JHMV-induced demyelination and fixed overnight in 4% paraformaldehyde at 4°C. Spinal cords were subsequently isolated and cryoprotected in 30% sucrose for 3–5 days. Thoracic and lumbar sections were embedded in OCT compound (Thermo Fisher Scientific) and frozen and stored at –80°C. Spinal sections (8 μm) were cut by cryostat and stained with Luxol fast blue (212170250, Thermo Fisher) along with hematoxylin (8495, ENG Scientific) and eosin (E511-25, Fisher Chemical). Demyelination was assessed by dividing the area of demyelinated white matter by total white matter, as previously described (75, 76). For immunofluorescence, spinal cord sections were blocked in 5% normal goat serum (005-000-121, Jackson ImmunoResearch) and 0.1% Tween-20 for 1 hour at room temperature. Sections were stained overnight at 4°C with primary antibody rabbit anti-MBP (1:1000; PA1050, Boster), followed by secondary antibody goat anti-rabbit conjugated with Alexa Fluor 488 (1:750; A11008, Invitrogen) for 2 hours at room temperature. Primary and secondary antibodies were diluted in 1% BSA and 0.1% Tween-20. Coverslips were placed on slides using Fluoromount-G with DAPI (00495952, Invitrogen). Sections were imaged with a Zeiss Axioscan 7 Microscope Slide Scanner using a ×20 objective.

### Flow cytometry and spectral cytometry.

Spinal cords and brains were harvested at peak disease or a specified time point and mechanically dissociated by passing through a 100 μm cell strainer. A 30%/70% Percoll gradient (catalog 17544501, Cytiva) was used to enrich mononuclear cells. Cells from collected spleens and lymph nodes (cervical, brachial, axillary, and inguinal) were passed through a 70 μm strainer, and erythrocytes were lysed by ammonium-chloride-potassium lysis buffer (150 mM NH_4_Cl, 10 mM KHCO_3_, 0.1 mM Na_2_EDTA). For intracellular staining, cells were cultured in complete medium (RPMI 1640, 10% FBS, 1% penicillin/streptomycin, Glutamax) supplemented with 1 μL/mL brefeldin A (555029, Golgiplug, BD), 50 ng/mL phorbol myristate acetate (P1585, Sigma-Aldrich), and 1 μg/mL ionomycin (I0634, Sigma-Aldrich) for 4 hours. Mouse I-A(b) MOG_38-49_ tetramers (GWYRSPFSRVVH) (77, 78) and control I-A(b) human class II-associated invariant chain peptide (CLIP) tetramers (PVSKMRMATPLLMQA) conjugated to allophycocyanin (APC) or phycoerythrin (PE) were synthesized by the NIH tetramer core facility. Cells were stained with control CLIP or MOG_38-49_ tetramers for 1 hour at 37°C prior to live/dead, surface, and intracellular staining. Cultured cells were fixed using Cytofix/Cytoperm (554714, BD Biosciences) according to manufacturer’s protocol, and stained for intracellular cytokines in Perm/Wash buffer (BD Biosciences).

Antibodies used for flow cytometry included CD45-Percp (30F11; 103129, Biolegend); CD11b-APC/Cy7 (M1/70; 101225, Biolegend); CD19-BUV661 (ID3; 612971, BD); CD19-FITC (103/CD19; 152404, Biolegend); NK1.1-PE-Cy5 (PK136; 108715, Biolegend); CD3e-BV605 (17A2; 100237, Biolegend); CD3e-FITC (145-2C11; 100305, Biolegend); TCRb-BV570 (H57-597; 109231, Biolegend); CD4-BV711 (RM4-5; 100557, Biolegend); CD4-BUV395 (GK1.5; 565974, BD); CD8a-APC (53-6.7; 100712, Biolegend); CD8a-BUV737 (53-6.7; 612759, BD); I-A/I-E-BUV805 (M5/144.15.2; 748844, BD); CD62L-PE-Cy7 (MEL-14; 25-0621-82, eBioscience); CD44-Percp-Cy5.5 (IM7; 45-0441-80, eBioscience); Ly-108-BUV563 (13G3; 7741436, BD); FR4-BUV496 (12A5; 750415, BD); CD73-V450 (Ty/23; 561544, BD); IL-23R-BV421 (12B2B64; 150907, Biolegend); CXCR6-BV711 (SA051D1; 151111, Biolegend); PD-1-APC/Fire810 (29F.1A12; 135251, Biolegend); IFN-γ–PE–Cy7 (XMG1.2; 25-7311-82, eBioscience); IFN-γ–APC (XMG1.2; 17-7311-81, eBioscience); IL-17a–BV605 (TC11-18H10.1; 506927, Biolegend); IL-17A-BV650 (TC11-18H10.1; 506929, Biolegend); TNF-α–FITC (MP6-XT22; 506304, Biolegend); and GM-CSF–PE/Dazzle594 (MP1-22E9; 505421, BioLegend). Flow cytometry samples were profiled using a BD Fortessa (BD Biosciences) (Figures 2–4) or an Aurora spectral flow cytometer (Cytek) (Figures 1 and 5). FlowJo software (BD Biosciences) was used for data analysis.

### Prepolarization profiling and mixed adoptive transfer.

We immunized 10-week-old C57BL/6 *Ocab^fl/fl^* and *Ocab^fl/fl^;CD4-Cre* littermates with MOG_35–55_ in CFA, as described earlier in Methods. After 14 days, spleen and lymph node (cervical, axillary, brachial, and inguinal) cells were isolated and cultured in complete medium (RPMI 1640, 10% FBS, 1% penicillin/streptomycin, Glutamax) supplemented with 1 μL/mL brefeldin A (Golgiplug; 555029, BD), 50 ng/mL phorbol myristate acetate (P1585, Sigma-Aldrich), and 1 μg/mL ionomycin (I0634, Sigma-Aldrich) for 4 hours. Cells were stained for surface markers, fixed using the Foxp3/Transcription Factor Staining Buffer Kit (00-5523-00, Thermo Fisher), and stained for intracellular markers (IFN-γ and IL-17).

### Th1 and Th17 polarization and adoptive-transfer C57BL/6 EAE.

We used 10–12 week-old female C57BL/6 littermate animals for all experiments. Donor animals were immunized with MOG_35–55_ in CFA, as described earlier in Methods. At 10–14 days after immunization, spleen and lymph node (cervical, axillary, brachial, and inguinal) cells were collected and placed into mixed culture with MOG peptide and cytokines at 5.0 × 10^6^ cells/mL. For Th1 conditions, complete medium was supplemented with 50 μg/mL MOG_35–55_, 6 ng/mL rmIL-6 (216-16, Peprotech), and 2 ng/mL recombinant mouse (rm) IFN-γ (315-05, Peprotech). For Th17 conditions, complete medium was supplemented with 50 μg/mL MOG_35–55_, 8 ng/mL rmIL-23 (1887-ML-010, R&D Systems), 10 ng/mL rmIL-1α (211-11A, Peprotech), and 10 μg/mL anti–IFN-γ (BE0055, BioXcell XMG1.2). After 4 days of respective culture, CD4^+^ T cells were purified using negative magnetic selection and resuspended in PBS at 1.5 × 10^6^ cells/mL. A 100 μL cell suspension was injected i.p. into age-matched male C57BL/6J recipient mice (The Jackson Laboratory, 000664) (~13 weeks old at time of disease induction). Clinical disease was scored for 15 days.

### JHMV.

*Ocab^fl/fl^* and *Ocab^fl/fl^*;CD4-Cre C57BL/6 mice were anesthetized using isoflurane. Mice were intracranially injected with 1500 PFU of JHMV (strain V34) suspended in 30 μL of Hank’s balanced salt solution (Thermo Fisher). Clinical disease was assessed for 21 days using a previously described scale (74). Mice were sacrificed at 7, 12, and 21 dpi to assess viral titers within brain homogenates using a plaque assay previously described (79). Spinal cords were taken on 12 and 21 dpi to assess demyelination by DAPI/MBP and by Luxol fast blue and hematoxylin and eosin histology, respectively.

### OCA-B–mCherry adoptive-transfer EAE.

OCA-B–mCherry reporter allele animals (31) were primed for 14 days with MOG_35–55_ in CFA, as described previously in Methods. After priming, spleen and lymph nodes were collected and CD4^+^ T cells were purified by magnetic negative selection (Miltenyi Bio kit and LS columns). Non–CD4^+^ T cells were removed from the LS column and placed on ice for subsequent in vitro culture. Purified CD4^+^ T cells were separated by OCA-B–mCherry expression (PE-Texas Red) using FACS on a BD Aria cell sorter. OCA-B–mCherry–positive and –negative CD4^+^ T cells were placed into mixed culture in complete medium with the previously purified non–CD4^+^ T cells at a 1:10 ratio (CD4^+^ to non-CD4^+^) at 1.0 × 10^6^ cells/mL. The culture was supplemented with 20 μg/mL MOG_35–55_ and 0.5 ng/mL IL-12p70 (210-12, Peprotech). After 2 days of culture, CD4^+^ T cells were again purified through negative magnetic selection. Purified CD4^+^ T cells from OCA-B–mCherry positive and negative cultures were resuspended in PBS at 8.8 × 10^6^ cells/mL. A 100 μL cell suspension was injected i.p. into age-matched male C57BL/6J recipients. Clinical disease was assessed for 24 days.

### Bulk RNA-Seq.

OCA-B–mCherry reporter mice (*n* = 9) were primed for 14 days with MOG_35–55_ in CFA, as we have described. After priming, lymph nodes (cervical, axillary, brachial, and inguinal) were isolated and mCherry-positive and -negative CD4^+^ T cells were collected by FACS. Total RNA, 3 groups (pooled, *n* = 3 mice each), was isolated using the Quick RNA micro kit (R1050, Zymo Research) with DNase treatment (79254, Qiagen). RNA concentration was measured with a Qubit RNA HS Assay Kit (Q32855, Fisher Scientific). RNA quality was evaluated with an RNA ScreenTape Assay (5067-5579 and 5067-5580, Agilent Technologies). Total RNA samples (5–500 ng) were hybridized with NEBNext rRNA Depletion Kit v2 (E7400, New England Biolabs) to diminish rRNA from the samples. Stranded RNA-Seq libraries were prepared as described using the NEBNext Ultra II Directional RNA Library Prep Kit for Illumina (E7760L, New England Biolabs). Purified libraries were qualified on a 4150 TapeStation (Agilent Technologies) using a D1000 ScreenTape assay (5567-5582 and 5067-5583, Agilent Technologies). The molarity of adapter-modified molecules was defined by quantitative PCR using the Kapa Biosystems Kapa Library Quant Kit (KK4824, Roche). Individual libraries were normalized to 5 nM. NovaSeq 150 × 150 bp sequencing libraries were chemically denatured and applied to an NovaSeq flow cell using the XP workflow (20043131, Illumina). After transfer to an Illumina NovaSeq 6000 instrument, 150 × 150 cycle paired-end sequencing was performed using a S4 reagent Kit v1.5 (20028312, Illumina). Between 8 million and 11 million reads were generated per sample and were aligned to the *GRCm38* mouse reference genome. Differential gene expression and pathway analysis were conducted using the *DESeq2* and *Enrichr* R packages.

### NOD-EAE.

Female littermate *Ocab^fl/fl^* and *Ocab^fl/fl^*;CD4-Cre animals (15–18 weeks old) backcrossed to a NOD strain background (32) were used for all experiments. Diabetic mice (blood glucose > 150 mg/dL) were excluded from the analysis. EAE was induced by two 100 μL s.c. injections, into both hind flanks, of 0.5 μmol/mL MOG_35–55_ peptide emulsified in incomplete Freuds adjuvant (77145, Thermo Fisher) supplemented with 4 mg/mL heat-killed *M*. *tuberculosis* (DF3114338, Fisher Scientific) to form CFA. Animals were given two 400-ng i.p. injections of *B*. *pertussis* toxin (81, List Biological Laboratories) on days 0 and 2. Clinical disease was assessed over 47 days using the same 5-point scale described previously under Clinical Scoring. Remission time points were determined to be between days 22 and 26; first relapse was determined to be between days 26 and 39.

### scRNA-Seq.

NOD.*Ocab^fl/fl^* and NOD.*Ocab^fl/fl^*;CD4-Cre animals were induced with EAE and monitored for disease progression. Brains and spinal cords were isolated on day 24 (remission) and day 33 (relapse), and processed for FACS sorting. Viable cells were sorted by CD3ε using a FACSAria (BD Bioscience). Cells from each condition were isolated from 3–4 mice and combined. Cells were processed using the 5′ 10X Genomics Chromium platform according to the manufacturer’s instructions. Paired-end high-throughput sequencing (*n* = 125 cycles) was performed using a NovaSeq instrument (Illumina). Sequencing reads were processed with the 10X Genomics CellRanger pipeline and further analyzed using the *Seurat* R package. Analysis of cells used a standard filtering protocol removing cells with unique feature counts of more than 5000 or less than 100, as well as cells with greater than 10% mitochondrial counts (indicative of dead cells). No more than 10% of total cells were removed by this process. Cells were subjected to unsupervised hierarchical clustering followed by UMAP to visualize clusters with similar gene expression and their relative positions. RNA velocity analysis was performed using velocyto.R, version 0.6, to model cell-state transitions using the guidelines provided in La Manno et al. (49) and the associated repository at https://github.com/velocyto-team/velocyto.R, Branch: HEAD, Commit ID: 83e6ed92c2d9c9640122dcebf8ebbb5788165a21. High-dimensional velocity vectors were graphed on the predefined Seurat UMAP projection using localized average grid projections. Pseudotime analysis was conducted using the *Seurat* and *Monocle3* packages. Integrated data were divided into subsets based on genotype, and *Monocle3* was used to calculate trajectory, order cells, and plot pseudotime for each condition.

### Statistics.

Multiple 2-tailed student’s *t*-tests were used to determine statistical differences in EAE clinical scores, and all error bars denote ± SEM unless otherwise noted. Two-way ANOVA was used to determine differences in weight loss throughout disease progression. GraphPad Prism software was used for all statistics and graphing. Two-tailed Student’s *t* tests were used to determine statistical differences in cell populations observed by flow cytometry, and all error bars represent ± SD unless otherwise noted.

### Study approval.

All animal experiments were performed in strict accordance with the NIH guide for the Care and Use of Laboratory animals and institutional guidelines for animal care at the University of Utah under approved protocol 00001553.

### Data availability.

All RNA-Seq, single-cell sequencing, and single-cell T cell V(D)J sequencing data associated with the findings of this study have been deposited to National Center of Biotechnology Information’s GEO database under accession code GSE243727. Data values for all graphs and values behind any reported means are reported in the Supporting Data Values file.

## Author contributions

EPH and DT conceived the study. EPH and ARS performed experiments. EPH, ARS, EMM, and BTS analyzed data. TEL, BTS, and DT provided critical resources and supervision for the study. EPH and DT wrote, reviewed, and revised the manuscript.

## Supplementary Material

Supplemental data

Supplemental table 1

Supplemental table 2

Supplemental table 3

Supplemental table 4

Supplemental table 5

Supporting data values

## Version 1 04/29/2025

In-Press Preview

## Version 2 07/01/2025

Electronic publication
