# Supplementary material for: OCA-B promotes pathogenic maturation of stem-like CD4+ T cells and autoimmune demyelination
Source: J Clin Invest. 2025 Apr 29;135(13):e187862. doi: 10.1172/JCI187862 (PMC12208553; doi:10.1172/JCI187862)
Supplement: Supplemental data [file jci-135-187862-s055.pdf]

Supplemental Information

OCA-B promotes pathogenic maturation of stem-like CD4+ T cells and autoimmune demyelination

Erik P. Hughes, Amber R. Syage, Elnaz Mirzaei Mehrabad, Thomas E. Lane, Benjamin T. Spike, Dean Tantin

Corresponding author: Dean Tantin, dean.tantin@path.utah.edu

Supplementary figures

Supplemental Figure 1

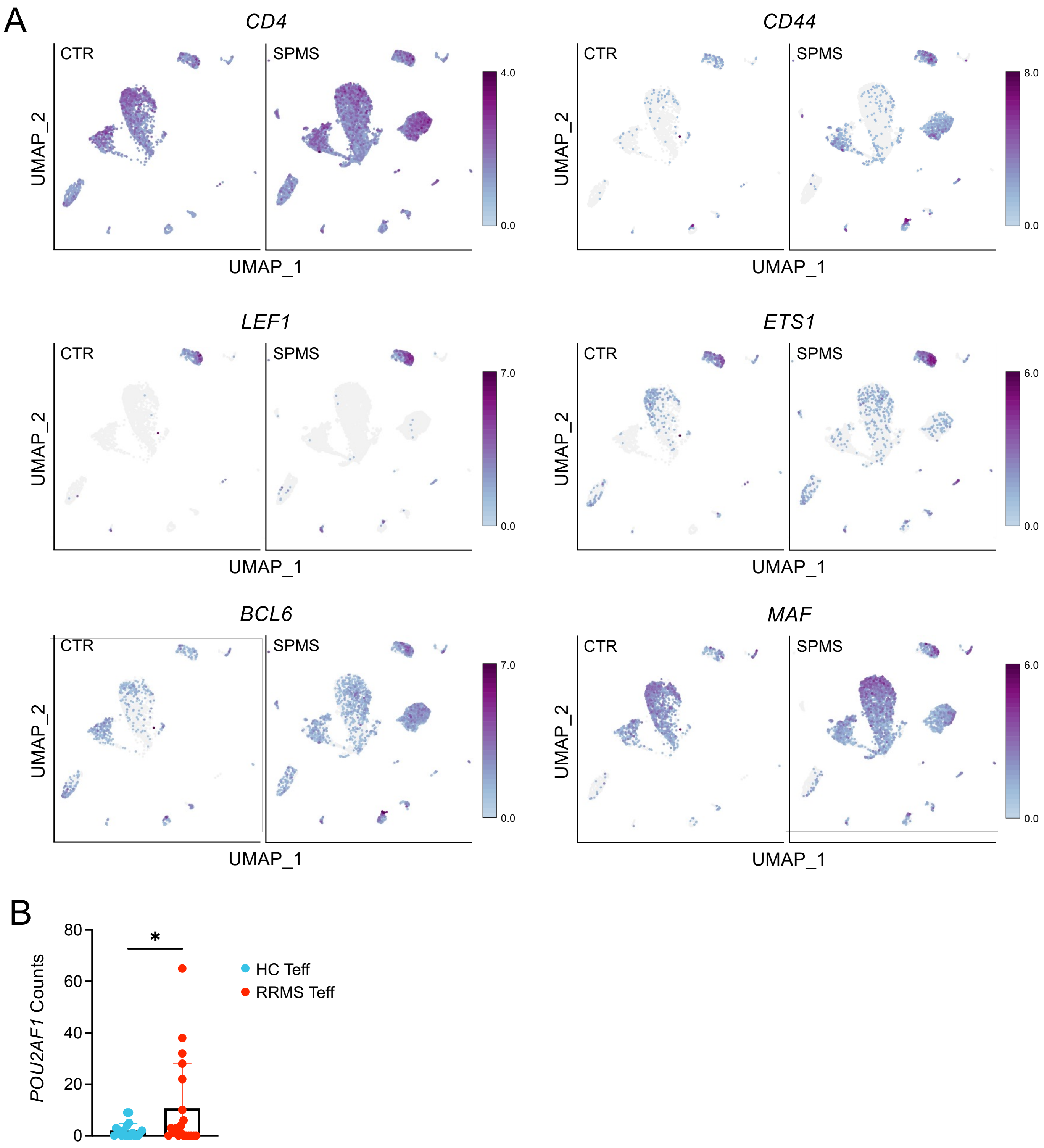

Supplemental Figure 1. Reclustered CD4$^+$ nuclei gene expression and analysis of peripheral blood CD4$^+$ T effector cell bulk RNAseq . (**A**) Additional feature plots of reclustered CD4$^+$ T cell nuclei from single-nucleus RNAseq data of secondary-progressive MS (SPMS) patient lesions and controls (McNair et al. 2025 Neuron 113:396) showing cluster gene expression of *CD4*, *CD44*, *LEF1*, *ETS1*, *BCL6*, and *MAF*. (**B**) Quantification of *POU2AF1* counts from bulk RNAseq data of CD4$^+$ T effector cells isolated from the peripheral blood of control (HC Teff) and MS (MS Teff) patients (Sumida et al. 2024 Sci. Trans. Med. 16:eapd1720). All data represent mean ±SD and Welch's T-test was used to determine statistical significance for data with unequal variance.

Supplemental Figure 2

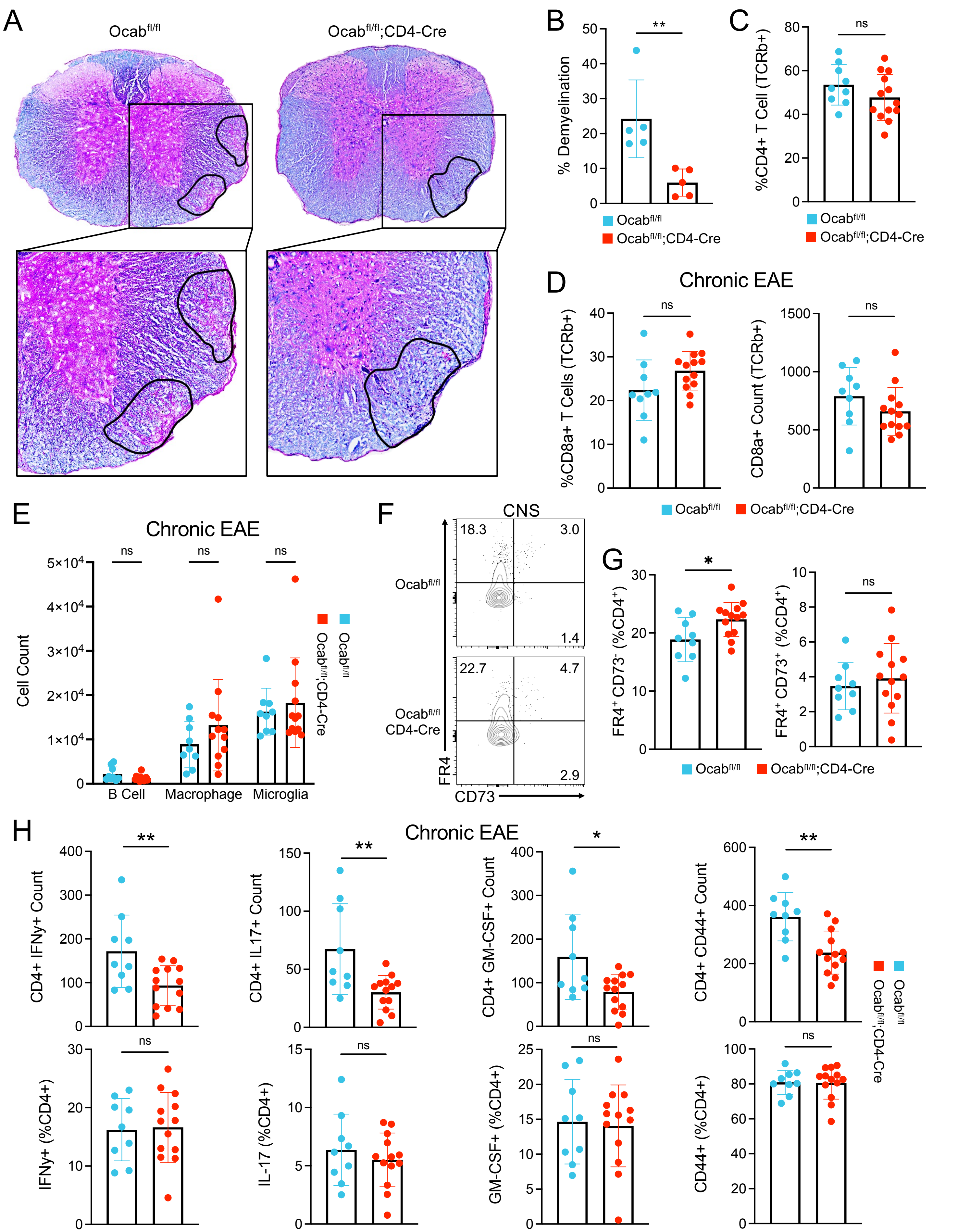

Supplemental Figure 2. Minimal differences in cytokine expressing CD4$^+$ T cell, CD8$^+$ T cell, B cell, macrophage or microglial cell counts at peak chronic EAE in OCA-B knockouts. (**A**) Representative spinal cord LFB/H&E staining of thoracic sections taken from mice 15 days after EAE induction. Areas of demyelination are outlined in black and marked by increased loss of LFB (blue) staining and visibility of H&E (pink) within the white matter. (**B**) Quantification of the % demyelination from LFB/H&E histology. Significance was ascribed by two-tailed student's t-test. (**C**) Quantification of the frequency of CNS infiltrating CD4$^+$ T cells between *Ocab*$^{fl/fl}$ and *Ocab*$^{fl/fl}$;CD4-Cre mice. Significance was ascribed by two-tailed student's t-test. (**D**) Quantification of frequency and count of CD8α positive T cells between *Ocab*$^{fl/fl}$ and *Ocab*$^{fl/fl}$;CD4-Cre mice. Significance was ascribed by two-tailed student's t-test. (**E**) Quantification of the number of B cells, macrophages and microglia. Significance was ascribed by two-tailed student's t-test. (**F**) Representative flow cytometry plots comparing the expression of FR4 and CD73 within CNS infiltrating CD4$^+$ T cells from *Ocab*$^{fl/fl}$ and *Ocab*$^{fl/fl}$;CD4-Cre mice. (**G**) Quantification of the frequency of CNS infiltrating FR4$^+$ CD73$^-$ and FR4$^+$ CD73$^+$ CD4$^+$ T cells within *Ocab*$^{fl/fl}$ and *Ocab*$^{fl/fl}$;CD4-Cre mice. Significance was ascribed by two-tailed student's t-test. (**H**) Quantification of frequency and count of IFNγ, IL-17, GM-CSF, and CD44 expressing CD4$^+$ T cells from *Ocab*$^{fl/fl}$ and *Ocab*$^{fl/fl}$;CD4-Cre mice. Significance was ascribed by two-tailed student's t-test. All data represent mean ±SD.

Supplemental Figure 3

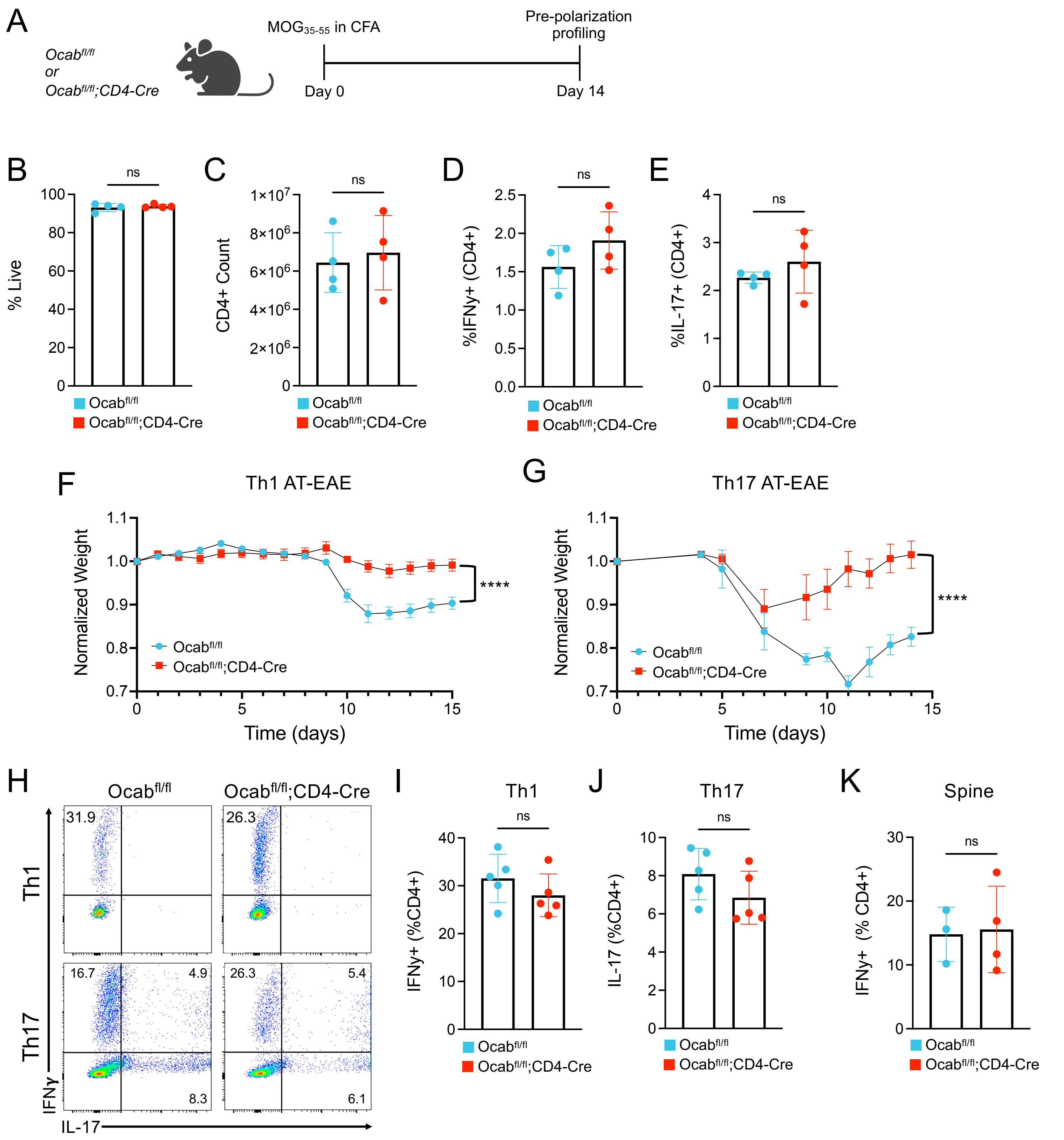

Supplemental Figure 3. Loss of OCA-B does not strongly alter initial Th1 or Th17 polarization in vitro. (**A**) Experimental schematic for assessing $CD4^+$ T cell differences between *Ocab*$^{fl/fl}$ and *Ocab*$^{fl/fl}$;CD4-Cre prior to *in vitro* Th1 or Th17 polarization. Lymph nodes (cervical, brachial, axillary, and inguinal) were harvested from *Ocab*$^{fl/fl}$ (n=4) and *Ocab*$^{fl/fl}$;CD4-Cre (n=4) mice following 14 days of MOG_{35-55}/CFA priming and analyzed by flow cytometry. (**B**) Frequency of viable cells isolated from *Ocab*$^{fl/fl}$ and *Ocab*$^{fl/fl}$;CD4-Cre mice. Significance was ascribed by two-tailed student's t-test. (**C**) Total $CD4^+$ T cell numbers isolated from *Ocab*$^{fl/fl}$ and *Ocab*$^{fl/fl}$;CD4-Cre mice. Significance was ascribed by two-tailed student's t-test. (**D**) Quantification of the frequency of IFNγ expressing $CD4^+$ T cells isolated from *Ocab*$^{fl/fl}$ and *Ocab*$^{fl/fl}$;CD4-Cre mice. Significance was ascribed by two-tailed student's t-test. (**E**) Quantification of the frequency of IL-17 expressing $CD4^+$ T cells derived from *Ocab*$^{fl/fl}$ and *Ocab*$^{fl/fl}$;CD4-Cre mice. Significance was ascribed by two-tailed student's t-test. (**F**) Normalized weights of recipient mice following adoptive transfer of *Ocab*$^{fl/fl}$ or *Ocab*$^{fl/fl}$;CD4-Cre Th1-polarized MOG-reactive $CD4^+$ T cells. Significance was ascribed by Two-way ANOVA. (**G**) Normalized weights of recipient mice following adoptive transfer of *Ocab*$^{fl/fl}$ or *Ocab*$^{fl/fl}$;CD4-Cre Th17 cells. Significance was ascribed by Two-way ANOVA. (**H**) Representative flow cytometry plots showing IFNγ and IL-17 expressing $CD4^+$ T cells from *Ocab*$^{fl/fl}$ (n=5) and *Ocab*$^{fl/fl}$;CD4-Cre (n=5) mice after Th1 and Th17 *in vitro* polarization. (**I**) Quantification of IFNγ expressing $CD4^+$ T cells after Th1 polarization. Significance was ascribed by two-tailed student's t-test. (**J**) Quantification of IL-17 expressing $CD4^+$ T cells after Th17 polarization. Significance was ascribed by two-tailed student's t-test. (**K**) Quantification of the frequency of IFNγ expressing $CD4^+$ T cells isolated from the spinal cords of Th17 adoptive transfer EAE recipient mice. Significance was ascribed by two-tailed student's t-test. Normalized weight variance is displayed as mean ±SEM. All other data represent mean ±SD.

Supplemental Figure 4

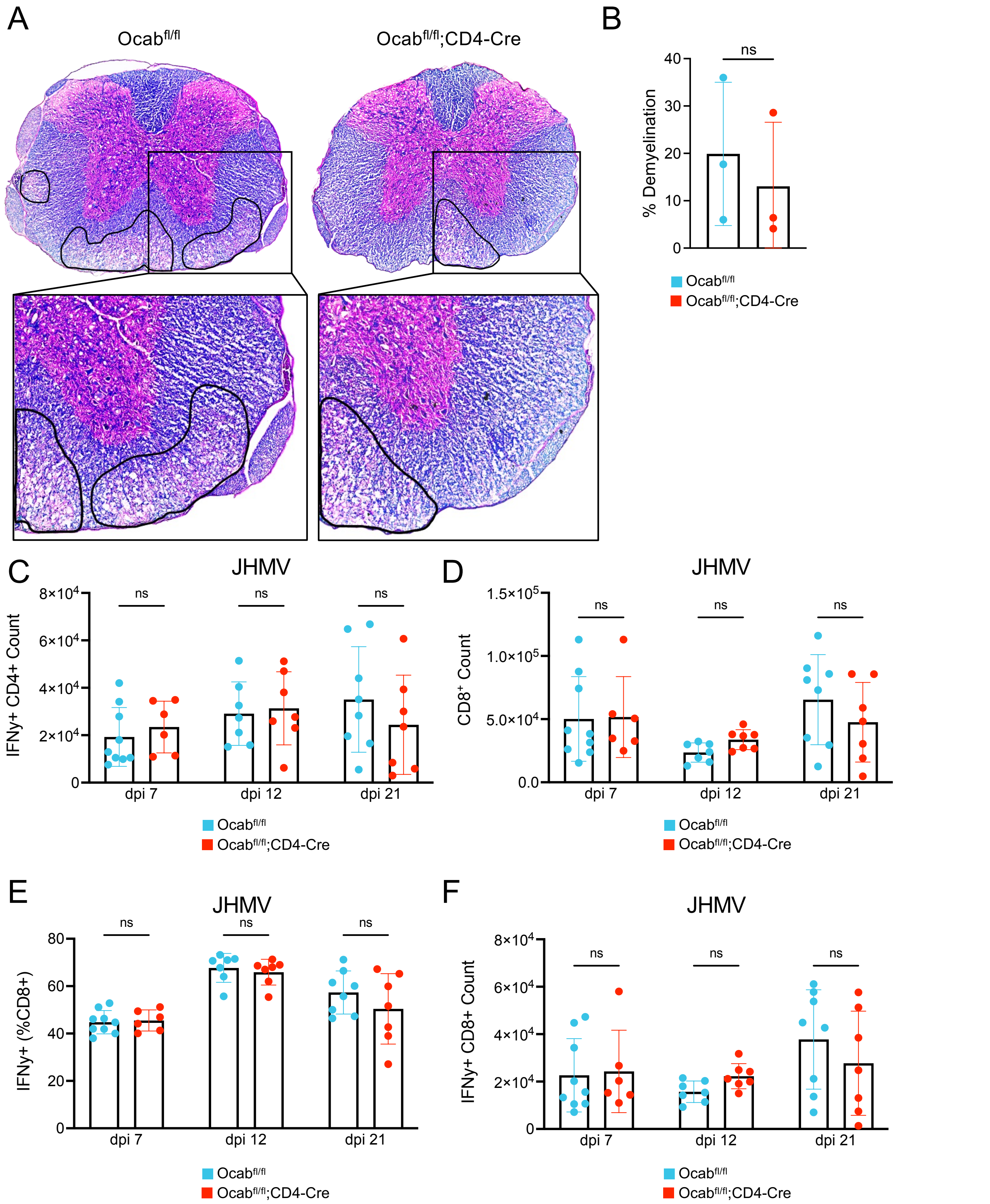

Supplemental Figure 4. Minimal changes in immune responses to intracranial JHMV infection in the absence of OCA-B. (**A**) Representative LFB/H&E histology of spinal cord sections at dpi 21. (**B**) Demyelination quantification of LFB/H&E histology at dpi 21. Significance was ascribed by two-tailed student's t-test. (**C**) Quantification of the number of IFNγ expressing $CD4^+$ T cells at dpi 7, 12 and 21. Significance was ascribed by two-tailed student's t-test. (**D**) Quantification of the number of $CD8^+$ T cells at dpi 7, 12 and 21. Significance was ascribed by two-tailed student's t-test. (**E**) Quantification of the frequency of IFNγ expressing $CD8^+$ T cells at dpi 7, 12 and 21. Significance was ascribed by two-tailed student's t-test. (**F**) Quantification of the number of IFNγ expressing $CD8^+$ T cells at dpi 7, 12 and 21. Significance was ascribed by two-tailed student's t-test. All data represent mean ±SD.

Supplemental Figure 5

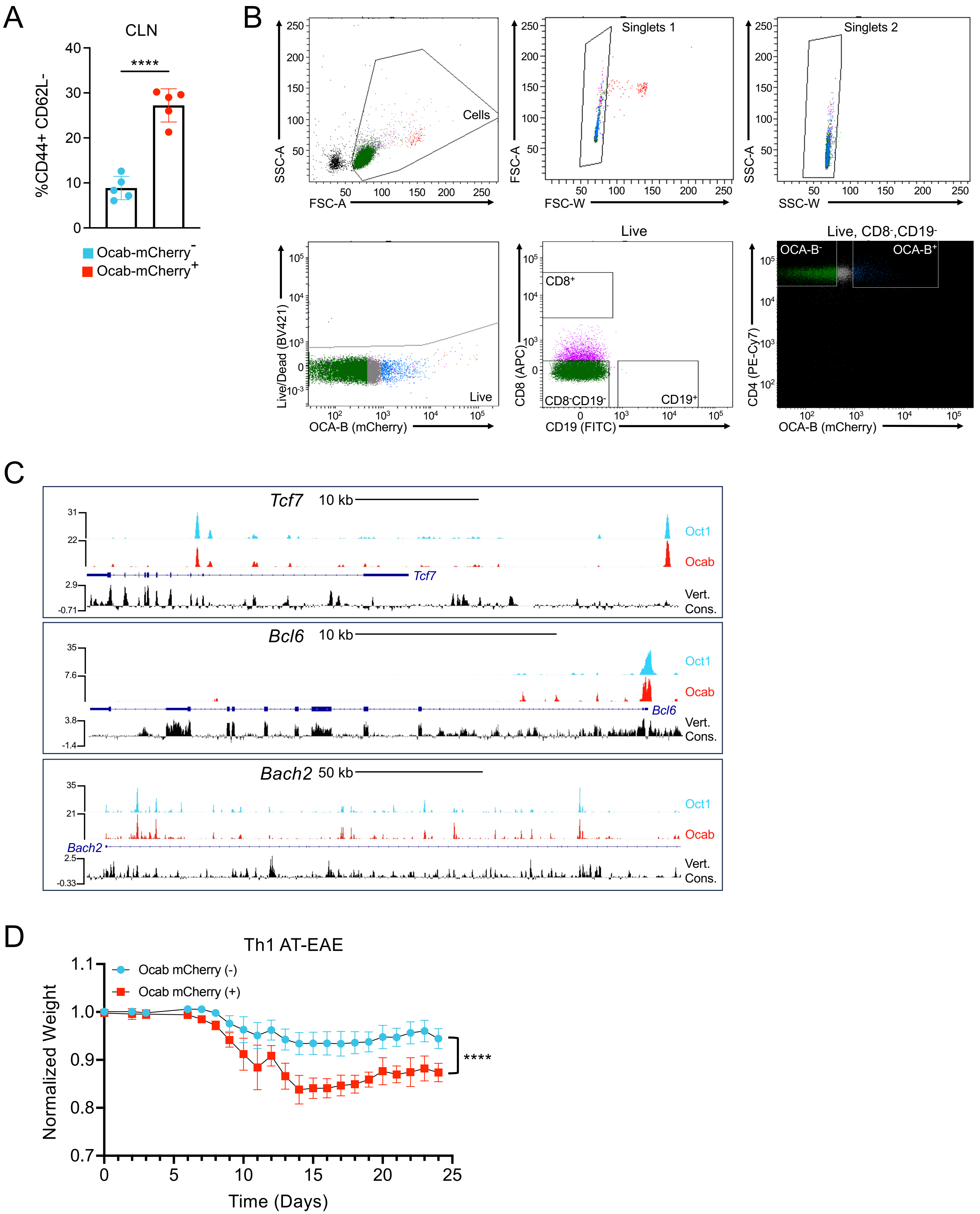

Supplemental Figure 5. Sorting OCA-B-mCherry reporter T cells and additional analysis of the effects of transferring OCA-B^{hi} versus OCA-B^{lo} CD4^{+} cells. (**A**) Quantification of the frequency of CD44^{+} CD62^{-} CD4^{+} T cells within the cervical lymph nodes of OCA-B-mCherry reporter mice at peak EAE by OCA-B-mCherry expression. Significance was ascribed by two-tailed student's t-test. (**B**) Gating strategy for sorting of OCA-B^{+} and OCA-B^{-} CD4^{+} T cells. (**C**) *mm39* ChIP-seq tracks showing peaks for Oct1 (blue), OCA-B (red), along with vertebrate conservation (black) at *Tcf7*, *Bcl6*, and *Bach2*. (**D**) Normalized weights of recipient mice following adoptive transfer of OCA-B-mCherry positive or negative Th1 cells. Significance was ascribed by Two-way ANOVA. Normalized weight is represented by mean ±SEM. All other data represent mean ±SD.

Supplemental Figure 6

A NOD.Ocab$^{fl/fl}$

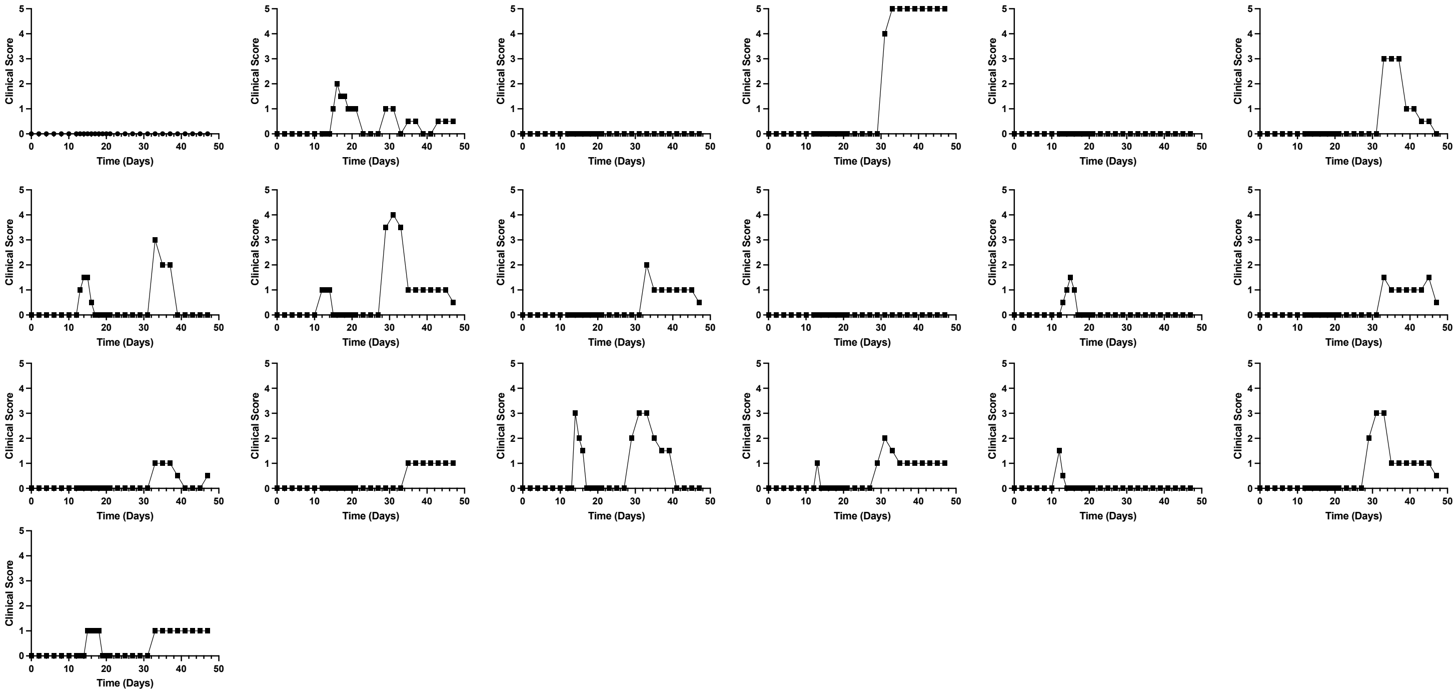

B NOD.Ocab$^{fl/fl}$;CD4-Cre

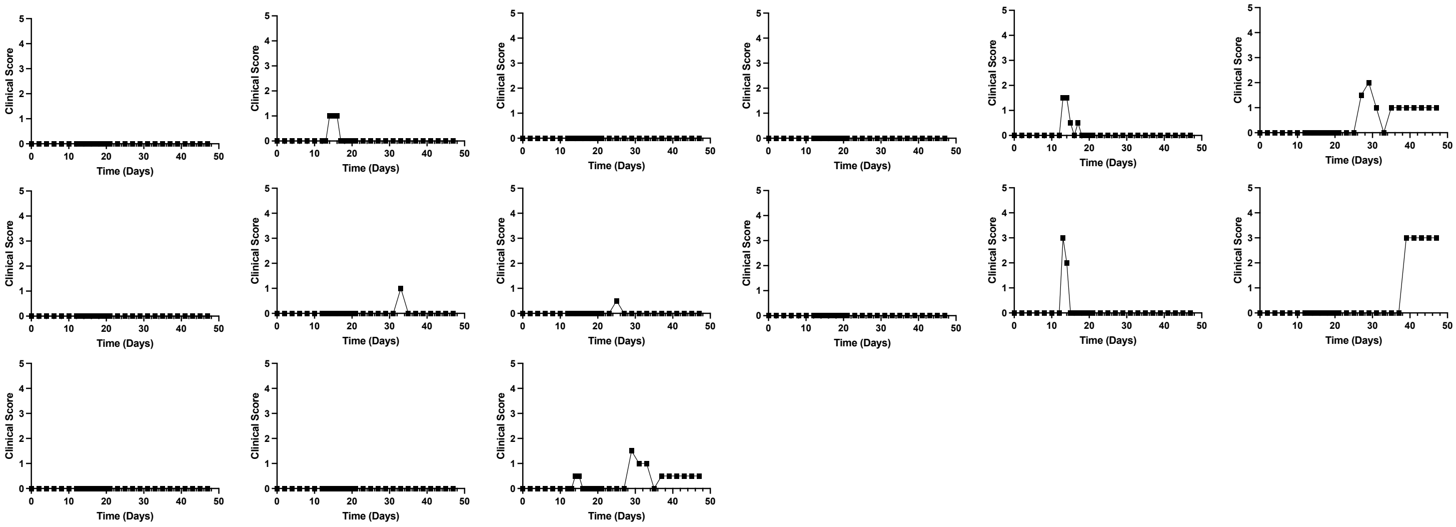

Supplemental Figure 6. Individual NOD mouse EAE clinical scores. (**A**) Clinical scores of individual NOD.*Ocab*$^{fl/fl}$ mice following EAE induction. (**B**) Clinical scores of individual NOD.*Ocab*$^{fl/fl}$;CD4-Cre mice following EAE induction.

Supplemental Figure 7

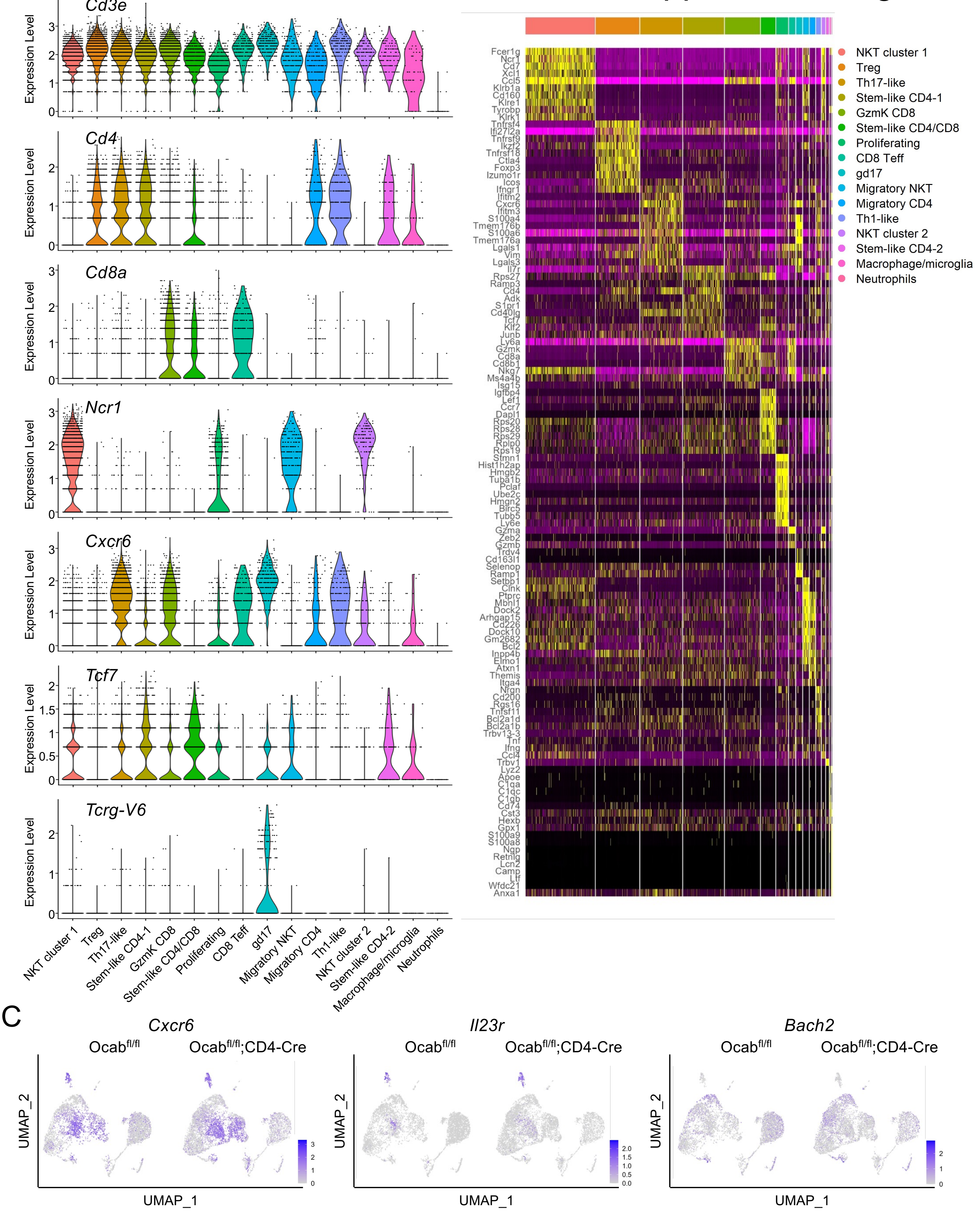

Supplemental Figure 7. Additional remission scRNA-seq cluster annotation, heat map of gene enrichments by cluster, and additional feature plots. (**A**) Violin plots showing cluster gene expression of *CD3e*, *Cd4*, *Cd8a*, *Ncr1*(NKp46), *Cxcr6*, *Tcf7*, and *Tcrg-V6* at EAE remission (**B**) Heatmap showing the top 10 genes expressed by cluster at remission EAE timepoint. (**C**) UMAP feature plots showing expression of *Cxcr6*, *Il23r*, and *Bach2* amongst clusters at remission.

Supplemental Figure 8

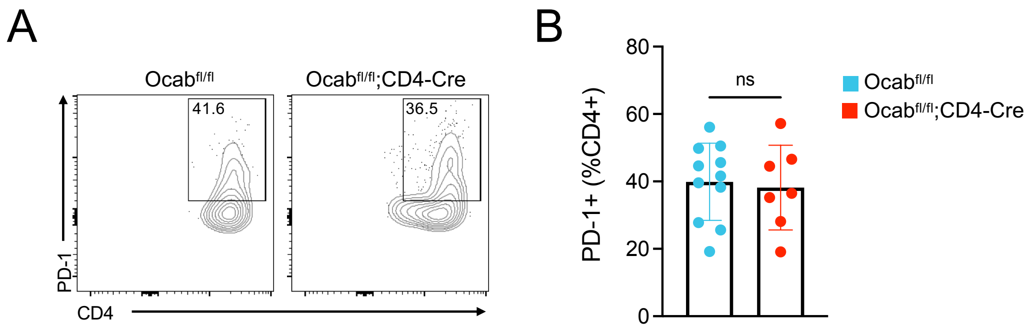

Supplemental Figure 8. EAE remission flow cytometry evaluating PD-1 expression in CNS infiltrating $CD4^+$ T cells. (**A**) Representative flow cytometry plots showing the frequency of PD-1 expressing $CD4^+$ T cells. (**B**) Quantification of the frequency of PD-1^+ $CD4^+$ T cells within the central nervous system of control and experimental mice at EAE remission. Significance was ascribed by two-tailed student's t-test. All data represent mean ±SD.

Supplemental Figure 9

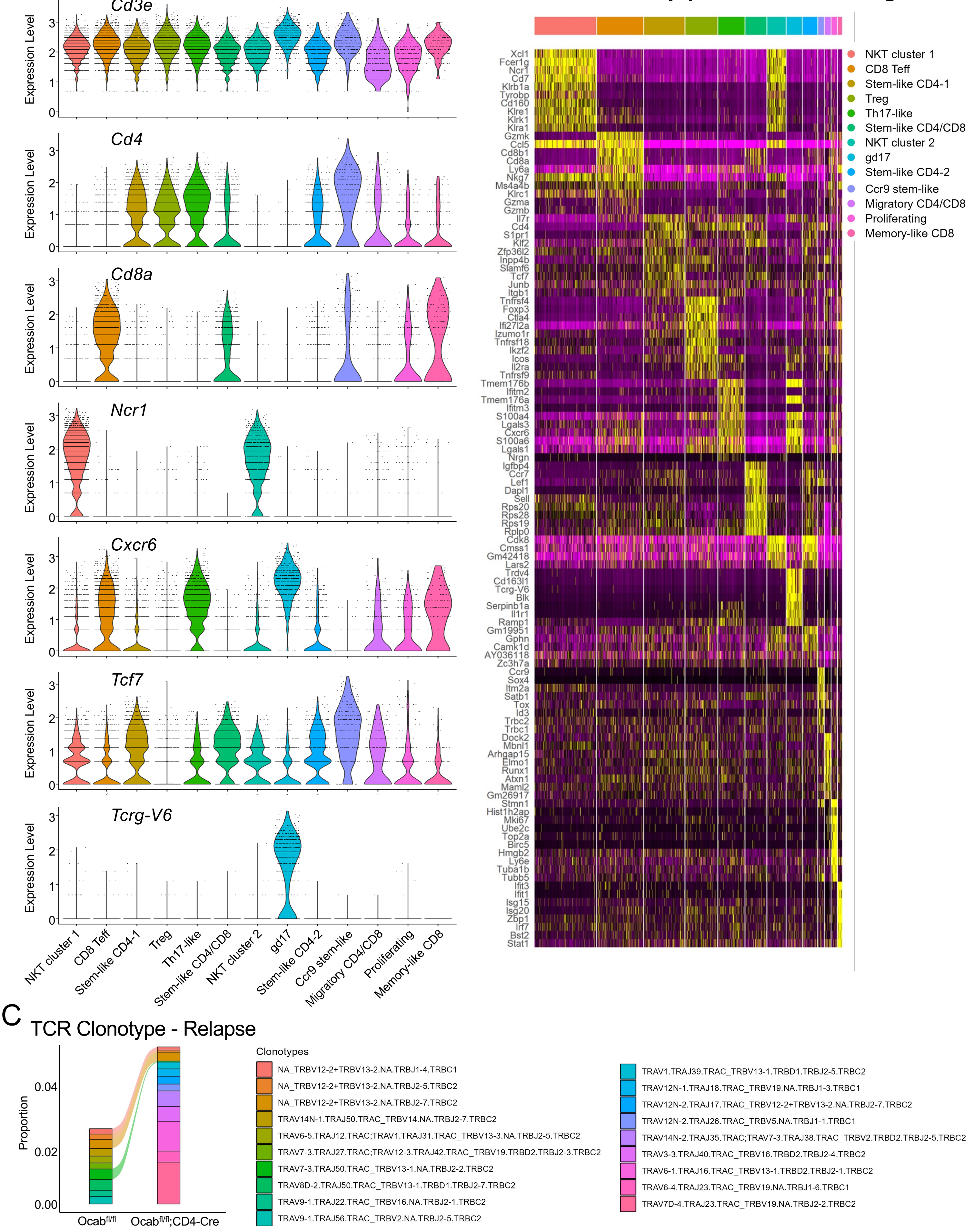

Supplemental Figure 9. scRNA-seq cluster annotation genes, heat map of NOD.EAE gene enrichments by cluster, and TCR clonotype overlap between conditions at the relapse timepoint. (**A**) Violin plots showing cluster gene expression of *CD3e*, *Cd4*, *Cd8a*, *Ncr1*(NKp46), *Cxcr6*, *Tcf7*, and *Tcrg-V6* at EAE relapse. (**B**) Heatmap showing the top 10 genes expressed by cluster at relapse EAE timepoint. (**C**) Alluvial plot showing top 10 TCR clonotype gene overlap between *Ocab*$^{fl/fl}$ and *Ocab*$^{fl/fl}$;CD4-Cre groups. (**C**) UMAP feature showing the expression of *CD4*, *CD8a, Tox*, *Sell*, *Ccr7*, and *Ccr4* amongst clusters at relapse timepoint.

Supplemental Figure 10

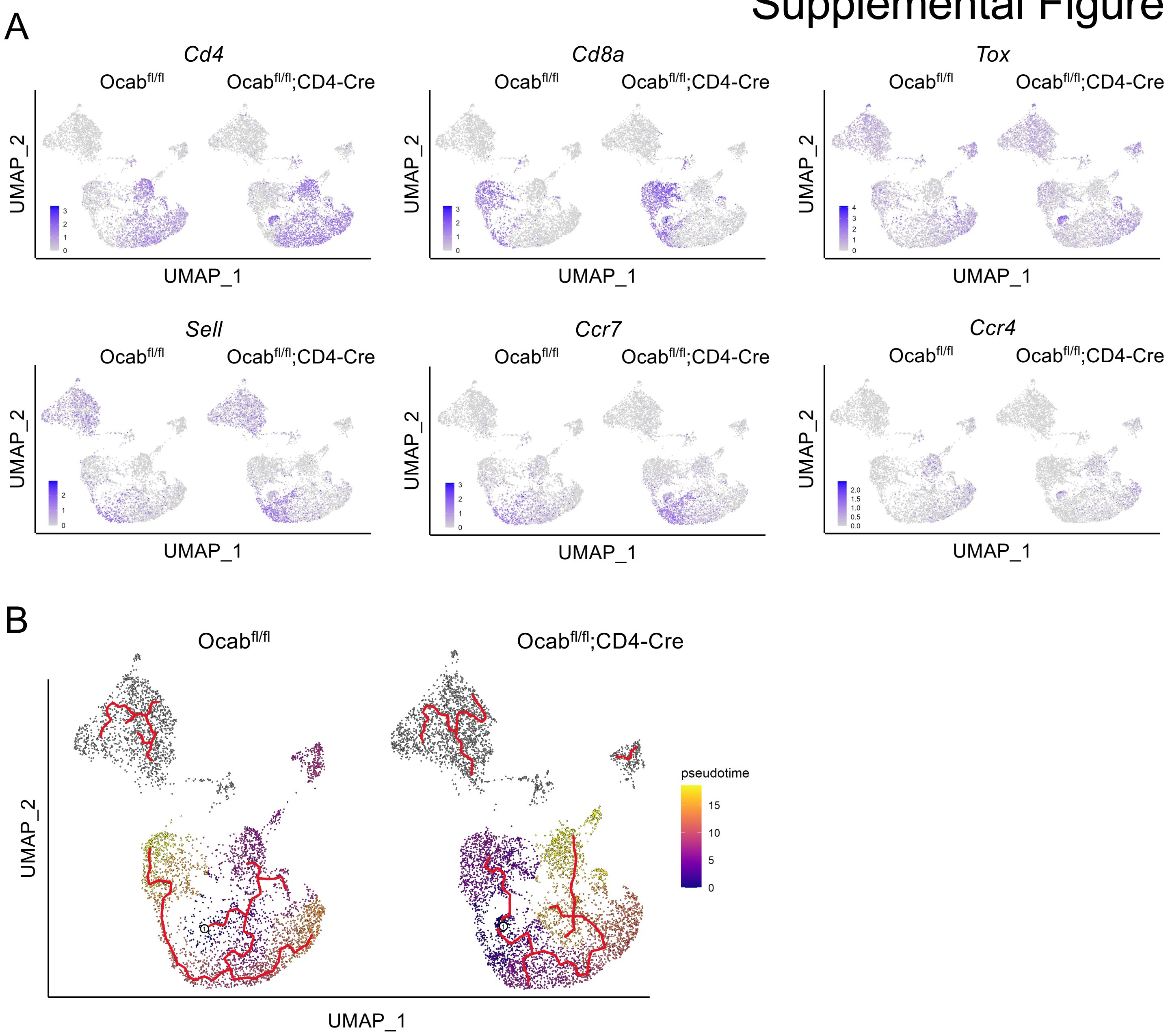

Supplemental Figure 10. Example relapse feature plots and pseudotime analysis of relapse single cell RNA seq. (**A**) UMAP feature showing the expression of *CD4*, *CD8a, Tox*, *Sell*, *Ccr7*, and *Ccr4* amongst clusters at relapse timepoint. (**B**) Pseudotime analysis of relapse single cell from root starting node at or near the *Ccr9*-expressing stem-like cluster.
